# Superior and efficient performance of cost-effective MIP-202 catalyst over UiO-66-(CO_2_H)_2_ in epoxide ring opening reactions

**DOI:** 10.1038/s41598-024-68497-2

**Published:** 2024-07-31

**Authors:** Mojtaba Bagherzadeh, Mohsen Chegeni, Arshad Bayrami, Mojtaba Amini

**Affiliations:** 1 Chemistry Department, Sharif University of Technology, PO Box, Tehran, 11155-3615 Iran; 2 Department of Chemistry, Research Center for Development of Advanced Technologies, Tehran, Iran; 3 Department of Inorganic Chemistry, Faculty of Chemistry, University of Tabriz, Tabriz, Iran

**Keywords:** Zr-based MOFs, Ring-opening reaction, Epoxides, MOF catalysis, MIP-202(Zr), UiO-66-(CO_2_H)_2_, Heterogeneous catalysis, Inorganic chemistry

## Abstract

This study explored the catalytic performance of two robust zirconium-based metal–organic frameworks (MOFs), MIP-202(Zr) and UiO-66-(CO_2_H)_2_ in the ring-opening of epoxides using alcohols and amines as nucleophilic reagents. The MOFs were characterized by techniques such as FT-IR, PXRD, FE-SEM, and EDX. Through systematic optimization of key parameters (catalyst amount, time, temperature, solvent), MIP-202(Zr) achieved 99% styrene oxide conversion in 25 min with methanol at room temperature using 5 mg catalyst. In contrast, UiO-66-(CO_2_H)_2_ required drastically harsher conditions of 120 min, 60 °C, and four times the catalyst loading to reach 98% conversion. A similar trend was observed for ring-opening with aniline –MIP-202(Zr) gave 93% conversion in one hour at room temperature, while UiO-66-(CO_2_H)_2_ needed two hours at 60 °C for 95% conversion. The superior performance of MIP-202(Zr) likely stems from cooperative Brønsted/Lewis acid sites and higher proton conductivity enabling more efficient epoxide activation. Remarkably, MIP-202(Zr) maintained consistent activity over five recycles in the ring-opening of styrene oxide by methanol and over three recycles in the ring-opening of styrene oxide by aniline. Testing various epoxide substrates and nucleophiles revealed trends in reactivity governed by electronic and steric effects. The results provide useful insights into tuning Zr-MOF-based catalysts and highlight the promise of the cost-effective and sustainable MIP-202(Zr) for diverse epoxide ring-opening reactions on an industrial scale.

## Introduction

In the realm of organic chemistry, epoxides have emerged as molecules of significant interest due to their three-atom ring structure that incorporates an oxygen atom^1^. This distinctive configuration provides epoxides with considerable ring strain and a polarized carbon–oxygen bond, making them exceptionally reactive. Such reactivity is beneficial across a broad spectrum of chemical reactions, facilitating the synthesis of diverse products including surfactants, polymers, resins, and pharmaceutical compounds^2^. Given their wide applicability, epoxides have catalyzed extensive research dedicated to exploring their chemical behavior and potential applications^3^. A challenge within epoxide chemistry is presented by ring-opening reactions^4^. These reactions are notable for their need of elevated temperatures and extended durations, emphasizing the demand for the development of catalysts that are not only efficient but also sustainable^5^. Among the critical reactions in this domain is the ring-opening by various nucleophiles, such as alcohols and amines, which leads to the formation of 1,2-disubstituted compounds^6,7^. In the context of drug synthesis, the reaction of epoxides with alcohols to yield β-alkoxy alcohols is of particular importance^8^. However, the relatively weak nucleophilic nature of alcohols necessitates the use of Brønsted or Lewis acid catalysts to achieve efficient reaction rates, often resulting in suboptimal selectivity^9^. Similarly, the synthesis of β-amino alcohols compounds extensively utilized in pharmaceuticals, including antimalarial drugs through the direct aminolysis of epoxides, highlights the need for catalytic methods that are both more efficient and selective, given the process's requirement for an excess of amines and high temperatures^10^. The quest for innovative catalysts capable of surmounting these hurdles is crucial for the advancement of epoxide chemistry. The catalysis landscape is diverse, employing both homogeneous and heterogeneous catalysts, each category bringing its own set of challenges to the fore. Homogeneous catalysts are extensively used due to their high efficiency, selectivity, and ability to operate under mild conditions. For instance, homogeneous catalysts such as Wilkinson’s catalyst RhCl(PPh_3_)_3_ and Grubbs’ catalyst RuCl_2_(P(Cy)_3_)_2_(=CHPh) have revolutionized industrial processes and synthetic chemistry due to their remarkable efficiency and selectivity in hydrogenation and olefin metathesis reactions, respectively. Despite these advantages, one of the main barriers to the broader industrial application of homogeneous catalysts is related to high costs, toxicity, and instability. These factors complicate their use in large-scale operations, often leading to complex and costly separation and recycling procedures^11–13^. In contrast, heterogeneous catalysts are limited because of complexities related to their synthesis, stringent purification requirements, harsh reaction conditions, and a lack of optimal selectivity^14^. Addressing these challenges is imperative for enhancing the efficiency of chemical reactions. Traditional catalysts, including alumina, silica, and alkaline metals, have been traditionally employed to augment the electrophilic characteristics of epoxides. Nevertheless, these catalysts frequently encounter issues such as extended reaction times, severe reaction conditions, and compromised selectivity and yield^15–17^. The elucidation of these limitations emphasizes the imperative need for research and development efforts focused on the identification and implementation of novel catalytic systems that promise to improve the field of epoxide catalysis, paving the way for more sustainable and effective synthetic methodologies.

In recent advancements within the realm of materials science, metal–organic frameworks (MOFs) have been considered as a groundbreaking class of materials, showing immense promise across a spectrum of applications, notably in catalysis. Distinguished by their remarkable attributes such as low density, extensive porosity, and expansive surface area, many MOFs have garnered significant attention in research. However, it is important to recognize that not all MOFs exhibit these properties uniformly. For example, while MOF-5, HKUST-1, and SIFSIX are notable for their extensive porosity and surface area, they suffer from limited chemical stability^18–21^. Among this diverse class, zirconium-based metal–organic frameworks (Zr-MOFs) stand out due to their adjustable pore size, minimal toxicity, and exceptional water stability, characteristics that are primarily attributed to the elevated oxidation state of Zr(IV)^22^. This specific state facilitates a pronounced charge density and bond polarization, fostering a strong interaction with carboxylate oxygen atoms prevalent in carboxylate-based Zr-MOFs. This interaction is perfectly compatible with Pearson’s hard/soft acid/base theory, positioning these Zr^4+^ MOFs as potent Lewis acids. Their efficacy has been particularly noted in facilitating the ring-opening reactions of epoxides, showcasing enhanced efficiency^23,24^. Despite significant research efforts, the quest to develop versatile and cost-effective catalysts capable of promoting ring-opening reactions across a broad range of nucleophiles remains a formidable challenge. Recent innovations, including the deployment of UiO-66 functionalized with thiophene-2-carboxamide^25^, copper-based MOF equipped with amine-derived tridentate ligands^26^, Fe_3_O_4_ nanoparticle-supported Mn (II)-azo Schiff complex^27^, and Lewis acidic trifluoromethylate ionic liquids have shown potential^28^. Nevertheless, these catalysts do not come without limitations which include prolonged reaction time, the necessity for high catalyst loadings, stability concerns, and environmental implications. Overcoming these hurdles to engineer versatile, economically feasible, and eco-friendly catalysts for the ring-opening of epoxides by both amines and alcohols would constitute a landmark achievement in organic chemistry. Such breakthroughs promise to facilitate the large-scale production of invaluable β-amino and β-alkoxy alcohols, thereby significantly benefiting the pharmaceutical sector and related industries.

This investigation delves into the catalytic capabilities of two zirconium-based MOFs, MIP-202(Zr) and UiO-66-(CO_2_H)_2_, in mediating the ring-opening reactions of epoxides using alcohol and amine nucleophiles. Introduced by Wang et al., in 2018, MIP-202(Zr) features a cost-effective, biocompatible framework comprised of aspartic acid and ZrCl_4_, serving as the organic linker and inorganic node, respectively. This framework is notable for its exceptional chemical stability across a wide pH range (0–12), outstanding water stability, and substantial proton conductivity^29^. On the other hand, UiO-66-(CO_2_H)_2_, synthesized from 1,2,4,5-benzenetetracarboxylic acid, provides mechanical robustness and thermal and water/pH stability. However, its higher production costs pose challenges for large-scale applications^30^. This study aims to highlight the potential of these Zr-MOFs in advancing the field of epoxide catalysis, paving the way for sustainable and efficient synthetic processes.

## Experimental

### Material and characterization techniques

The Supplementary Information file provides details on the materials and characterization techniques used in this study.

### Preparation of MIP-202(Zr) (MIP)

**Figure 1:**
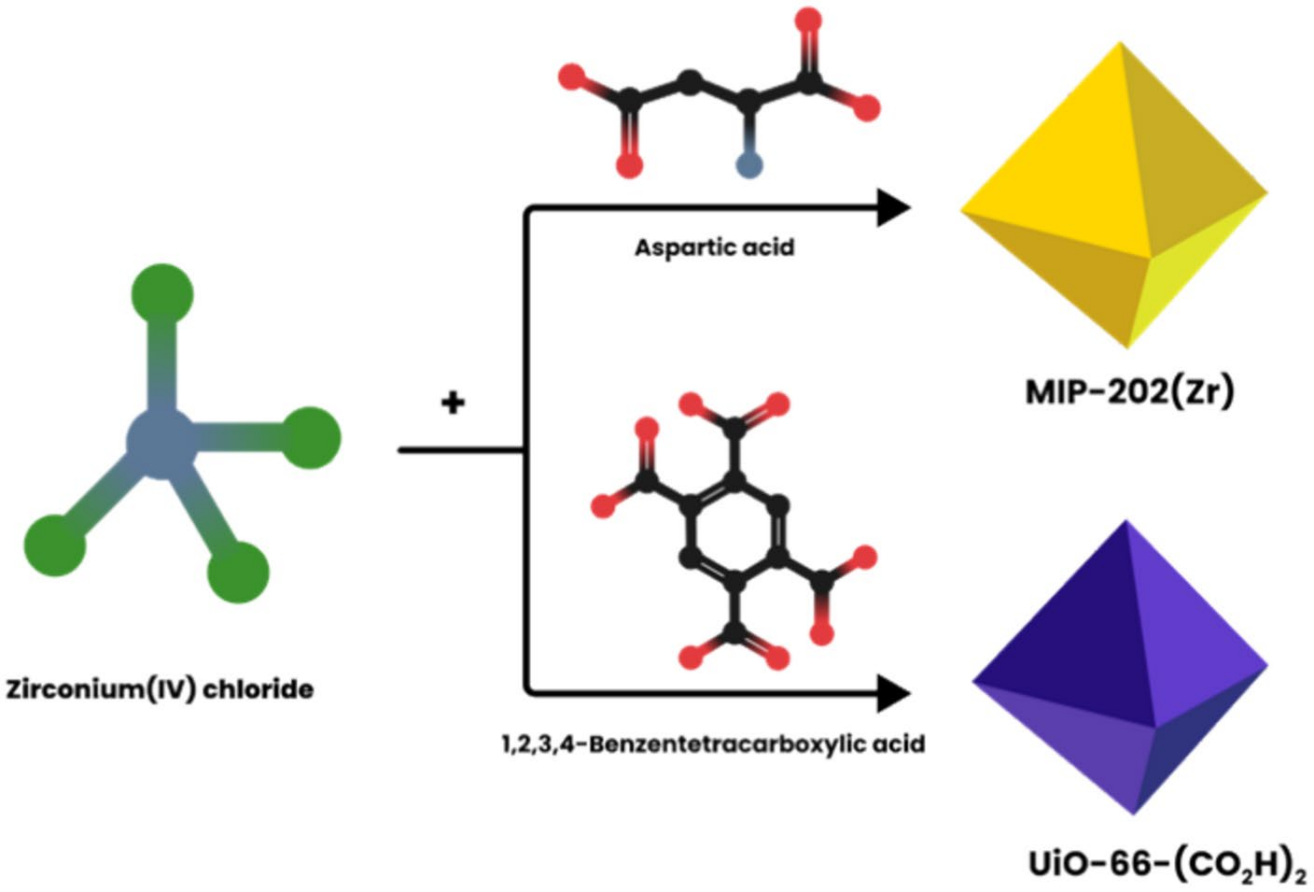
MIP-202 and UiO-(CO_2_H)_2_ preparation steps.

The synthesis of the MIP-202 MOF was conducted based on the protocol described by Wang et al.^29^, with slight modifications. Initially, 2 g of l-aspartic acid (15 mmol) was combined with 5 ml of distilled water in a 25 ml round-bottom flask. This mixture underwent sonication for 20 min to ensure uniform distribution of the contents. Following this, ZrCl_4_ (1.66 g; 7.1 mmol) was gradually added to the mixture in several steps, resulting in a colorless and transparent aqueous solution. To incorporate any residual solids adhering to the flask walls into the solution, an additional 5 ml of distilled water was used. The solution was then subjected to a reflux system at 120 °C for 1 h. After cooling, the formed white-colored precipitate was isolated from the mixture and subsequently immersed in 80 °C water for further purification. This step aimed to eliminate any unreacted linker materials. The purified product was collected through centrifugation, washed with water and ethanol, and then dried at 80 °C for 12 h, yielding the desired MIP (Fig. 1). The product synthesized using this method is termed MIP.

To enable comparison, we also synthesized MIP-202 using the hydrothermal method described by Tao et al., where the reaction was conducted in an autoclave. The product obtained from this method is referred to as MIP-a^31^.

### Preparation of UiO-66-(CO_2_H)_2_) (UiO)

The synthesis of the UiO-66-(CO_2_H)_2_ MOF was meticulously carried out following the procedures outlined in previous literature^30^. To start, a mixture of 1,2,3,4-benzenetetracarboxylic acid (0.65 g; 2.5 mmol) and ZrCl_4_ (0.6 g; 2.5 mmol) was prepared in a blend of distilled water (20 ml) and acetic acid (10 ml). This mixture underwent a thorough sonication process for 20 min, ensuring even distribution of the reactant materials. The sonicated mixture was then placed under reflux at 110 °C for a period of 24 h, resulting in the formation of a white-colored precipitate. The sediment was efficiently separated from the mixture using centrifugation, followed by thorough rinsing with ethanol and distilled water to remove any impurities. Subsequently, to activate the compound, the product was immersed in methanol for three days in succession, with a final step of maintenance in acetone. Any remaining solids were carefully rinsed off, and the final product was dried under vacuum conditions at 70 °C for 12 h (Fig. 1).

### General procedure for epoxide ring-opening reaction by alcoholic nucleophiles

The process for opening epoxide rings using alcohol nucleophiles is carried out batchwise. First, 1 mmol of epoxide is combined with 0.5 ml alcohol in a round bottom flask set up with a magnetic stir bar. The alcohol functions as both the solvent and nucleophile. Next, catalytically effective amounts of MOFs are added: 5 mg (0.003 mmol) of MIP or 20 mg (0.009 mmol) of UiO. The MIP-catalyzed reaction proceeds at room temperature, while 40–60 °C is necessary to drive the UiO-catalyzed process. Reaction progress over time is monitored by gas chromatography to quantify kinetics. After the reaction finishes, the product mixture is extracted into ethyl acetate and dried over magnesium sulfate. Proton NMR spectroscopy confirms product formation through characteristic signals. Finally, the MOF catalyst is recovered by centrifugation, washed with water and ethanol, and dried at 70 °C for 12 h to enable reuse^32,33^.

### General procedure for epoxide ring-opening reaction by amine nucleophiles

Here, too, the procedure goes in the same direction as the alcohol-based opening. Nonetheless, certain changes need to be incorporated. Instead of using 0.5 ml of alcohol as a reactant and solvent, 1 mmol of amine nucleophile which is added to 1 mmol epoxide substrate is used. The catalyst amounts are also modified: We add 15 mg (0.01 mmol) MIP and 20 mg (0.009 mmol) UiO. With no solvent to consider, these quantities reflect the tailored requirements of the amine system. Reaction progress tracking, product extraction into ethyl acetate, catalyst recovery, and product characterization steps mirror the previous procedure. This streamlined approach maintains the underlying ring-opening mechanism while optimizing conditions for amine nucleophile compatibility. By removing excess solvent and tuning catalyst levels, it enables efficient access to β-amino alcohols in an operationally simple, catalyst-friendly format^34–36^.

## Results and discussion

### Characterization of MIP-202(Zr)

**Figure 2:**
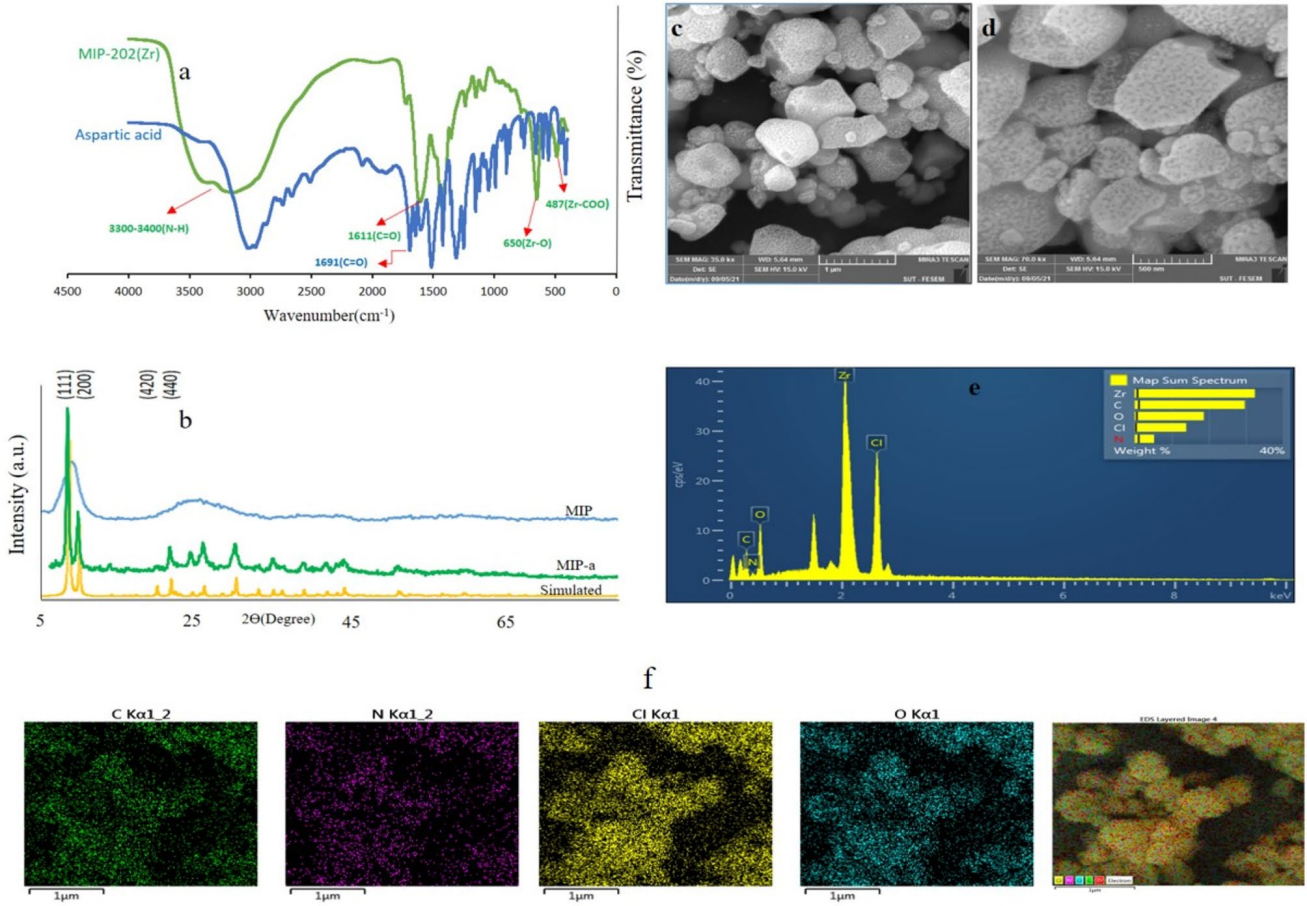
Characterization of MIP: (**a**) FT-IR spectra of MIP and aspartic acid; (**b**) PXRD patterns of the simulated and as-synthesized MIP and MIP-a; (**c** and **d**) FE-SEM images of MIP-202; (**e** and **f**) EDX elemental spectrum and elemental mapping images of MIP-202.

As illustrated in Fig. 2a, the Fourier transform infrared (FT-IR) spectrum of the freshly synthesized MIP MOF shows a band at 3300 cm^−1^, which is related to the presence of the NH functional group in the aspartic acid linker. Moreover, the spectrum includes two separate peaks at 1436 and 1611 cm^−1^, attributed to the C‒O symmetric and asymmetric stretching vibrations of the aspartic acid linker, respectively. Furthermore, the bands observed at 487 and 650 cm^−1^ are related to Zr‒O and Zr‒COO bonds, respectively. An important observation is the shift in the ν(C=O) absorption band for the MOF (1611 cm^−1^) compared to the free linker (1691 cm^−1^), which results from the formation of the Zr‒C=O bond and the reduction of electron density from C=O. This observation provides complementary evidence of the MOF's successful formation.

The simulated powder X-ray diffraction (PXRD) pattern of the MIP-202 (Fig. 2b) shows characteristic peaks at 2θ = 8.1°, 9.9°, 20°, and 21.8°, corresponding to the (111), (200), (420), and (440) planes, respectively^37^. In comparison, the experimental PXRD pattern of the as-synthesized MIP shows broader peaks, indicating lower crystallinity. Additionally, the PXRD pattern of MIP-a, synthesized through the hydrothermal method by Tao et al.^31^., shows well-defined peaks at 2θ = 8.1°, 9.9°, 20°, and 21.8°, closely matching the simulated pattern and confirming its good crystallinity. Despite slight peak broadening, MIP-a aligns well with the simulated pattern. In contrast, MIP exhibits significant peak broadening, suggesting more pronounced crystal defects and lower crystallinity.

Observations from field emission scanning electron microscopy (FE-SEM), shown in Fig. 2c and d, reveal that MIP nanoparticles exhibit a rough spherical morphology with a maximum distribution size of 500 nm (Fig. S3, Supplementary Information file). Previous studies have shown that the existence of H_3_(CH)N^+^ groups on the coordinated aspartic acid linkers, along with their strong ionic interactions with the chloride ions (the counter-anion from the metal source, ZrCl_4_), is held accountable for the Cl peak observed in the energy-dispersive X-ray (EDX) spectrum (Fig. 2e)^29^. One must take into account that the chloride anions were not removed from the MIP pores during the rinsing process with water and ethanol. Figure 2f illustrates the elemental distribution of the sample. This comprehensive characterization proves the successful synthesis and desired structural traits of the MIP MOF. Additionally, The N_2_ adsorption–desorption isotherms reveal that MIP-202(Zr) exhibits a relatively low specific surface area of 49.62 m2/g and a small pore size of 0.63 nm (Fig. S5 in the Supplementary Information).

### Characterization of UiO-66-(CO_2_H)_2_

**Figure 3:**
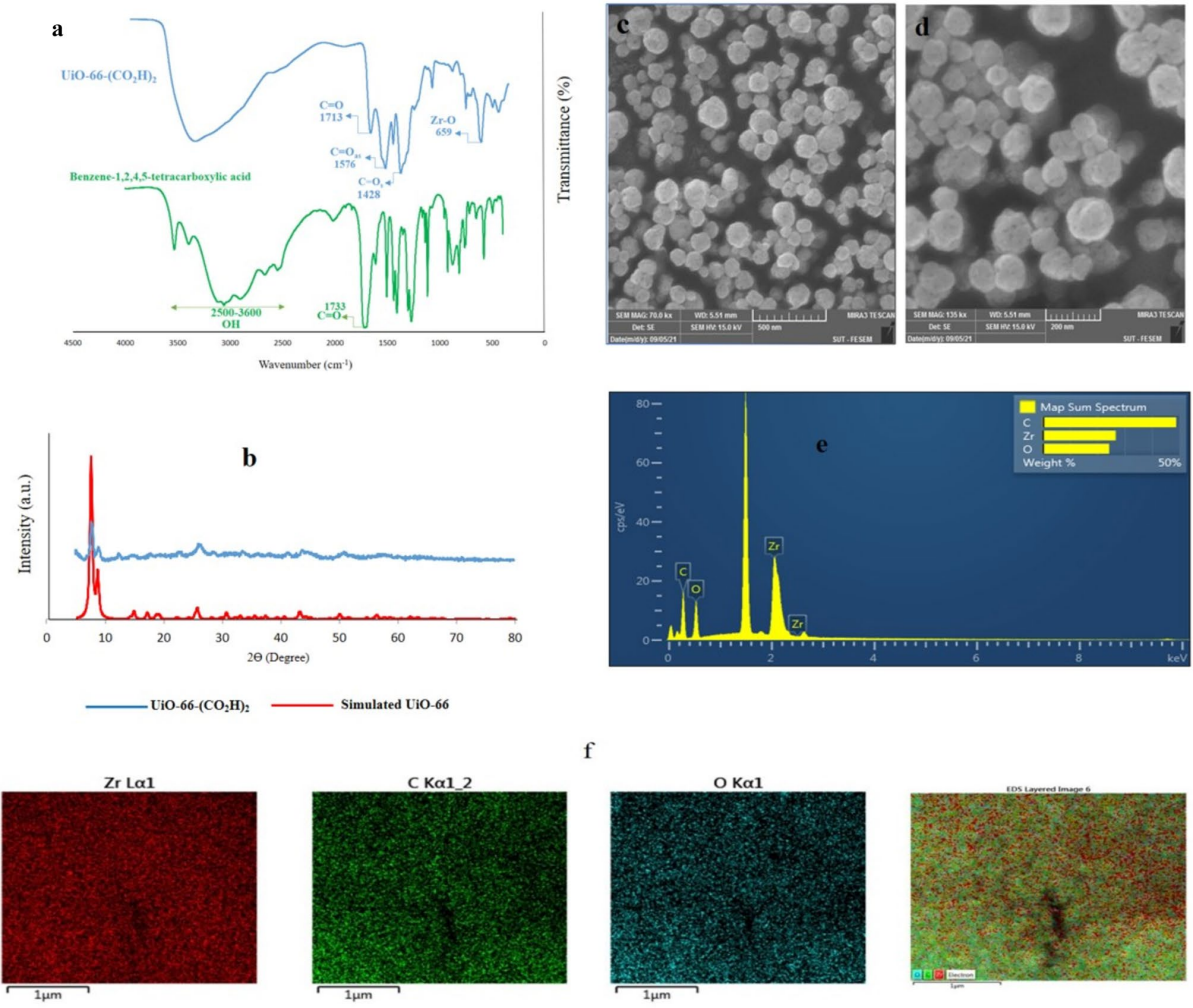
Characterization of UiO: (**a**) FT-IR spectra of UiO-66-(CO_2_H)_2_ and 1,2,3,4-benzenetetracarboxylic acid; (**b**) PXRD patterns of the simulated and as-synthesized UiO-66-(CO_2_H)_2_; (**c** and **d**) FE-SEM images of UiO-66-(CO_2_H)_2_; (**e** and **f**) EDX elemental spectrum and mapping images of UiO-66-(CO_2_H)_2_.

The FT-IR spectroscopy analysis of UiO-66-(CO_2_H)_2_, as depicted in Fig. 3a, reveals distinct peaks at 1574 and 1428 cm^−1^. These peaks are responsible for the asymmetric and symmetric stretching vibrations of COO^-^ groups of the linker, respectively. Additionally, the peak at 1713 cm^−1^ is attributed to the carbonyl groups of the uncoordinated COOH groups from ligand. The absorption band located at 659 cm^−1^ corresponds to ν(Zr‒O) vibrations, confirming the successful formation of the UiO-66-(CO_2_H)_2_ structure. Moreover, a noticeable shift in the ν(C=O) group from 1733 cm^−1^, which corresponds to the carbonyl group of the linker, to 1574 cm^−1^ in the MOF indicates the effective coordination of organic linkers to the Zr centers. This shift is attributed to the weakening of C=O bonds upon coordination with Zr, resulting in a lower wavenumber. The broad peak observed in the FTIR spectrum between 2500 cm^−1^ and 3600 cm^−1^ is attributed to the overlapping contributions from uncoordinated carboxyl groups (–COOH), adsorbed water molecules, and residual solvents. This combination results in the observed broadening.

The PXRD patterns, as presented in the simulated data, exhibit characteristic peaks at 2θ values of 7.4°, 8.5°, 12.1°, 14.2°, 17.1°, 22.3°, 25.7°, 31.1°, and 33.1°. These peaks correspond to the (111), (002), (113), (004), (115), (224), (046), and (137) crystal planes, respectively^38^. The observed peaks in the PXRD pattern of the as-synthesized UiO-66-(CO_2_H)_2_ match these characteristic peaks, confirming the formation of the intended MOF (Fig. 3b).

Further morphological and elemental analysis of the synthesized MOF is presented through FE-SEM images and EDX spectra, shown in Fig. 3c, d. The FE-SEM images reveal that the UiO-66-(CO_2_H)_2_ nanoparticles exhibit a spherical morphology with a maximum distribution size of 200 nm (Fig. S4, Supplementary Information file).The EDX spectrum and corresponding mapping images provide insights into the elemental composition and distribution within the nanoparticles, aligning with previously reported analyses in the literature (Fig. 3e and f)^39^. Additionally, the N_2_ adsorption–desorption isotherms reveal that UiO-66-(CO_2_H)_2_ (Fig. S6, Supplementary Information file) has a specific surface area of 259 m^2^ g^−1^ and a pore size of 8.442 nm. This comprehensive characterization confirms the successful synthesis and expected structural features of the UiO-66-(CO_2_H)_2_ MOF.

### Catalytic activity evaluation of the Zr-based MOFs in the epoxide ring-opening reactions by alcohol nucleophiles

The catalytic activity of the MOFs was explored in epoxide ring-opening reactions using an alcoholic nucleophile, with the reaction between styrene oxide and methanol serving as the model (Table 1). Initially, the influence of various parameters such as catalyst amount, reaction time, temperature, and solvent choice was examined for the MIP-catalyzed reaction. In the absence of the catalyst at room temperature, almost no product formation was observed even after 24 h (Table 1 entry 17). However, a significant increase in reaction conversion was noted when the MIP catalyst amount was raised to 0.3 mol%, reaching substantial conversion within just 25 min. Further increase in catalyst quantity did not reduce the reaction time (Table 1 entry 7), establishing 0.3 mol% as the optimal catalyst amount.

**Table 1:** A comparative evaluation of UiO and MIP catalysts highlights the impact of catalyst amount, temperature, time, and solvent on the styrene ring-opening reaction.


Entry	Catalyst	Mol% of catalyst^a^	Solvent	Time (min)	Temperature (°C)	Conversion (%)	Selectivity (%)
1	MIP	0.15	MeOH^b^	10	r.t	82	99
2	MIP	0.15	MeOH	20	r.t	87	99
3	MIP	0.3	MeOH	10	r.t	91	99
4	MIP	0.3	MeOH	20	r.t	97	99
5	MIP	0.3	MeOH	25	r.t	99	99
6	MIP-a	0.3	MeOH	25	r.t	70	99
7	MIP	0.6	MeOH	25	r.t	99	99
8	MIP	0.3	Water	25	r.t	60	25
9	MIP	0.3	Toluene	25	r.t	3	99
10	MIP	0.3	CH_3_CN	25	r.t	2	99
11	MIP	0.3	n-hexane	25	r.t	0	0
12	MIP	0.3	DCM	25	r.t	5	99
13	MIP	0.3	THF	25	r.t	4	99
14	MIP	0.3	EtOAc	25	r.t	6	99
15	MIP	0.3	Without solvent	25	r.t	27	99
16	No catalyst	0	MeOH	25	r.t	0	0
17	No catalyst	0	MeOH	24 h	r.t	2	99
18	UiO	0.225	MeOH	60	r.t	33	99
19	UiO	0.675	MeOH	60	r.t	42	99
20	UiO	0.9	MeOH	60	r.t	48	99
21	UiO	0.9	MeOH	90	r.t	60	99
22	UiO	0.9	MeOH	120	r.t	65	99
23	UiO	0.9	MeOH	120	50	83	99
24	UiO	0.9	MeOH	120	60	98	99
25	UiO	1.35	MeOH	120	r.t	73	99
26	UiO	1.35	MeOH	120	60	98	99
27	UiO	0.9	Water	120	60	57	99
28	UiO	0.9	Toluene	120	60	5	99
29	UiO	0.9	CH_3_CN	120	60	1	99
30	UiO	0.9	n-hexane	120	60	0	99
31	UiO	0.9	DCM	120	60	3	99
32	UiO	0.9	THF	120	60	2	99
33	UiO	0.9	EtOAc	120	60	6	99
34	UiO	0.9	without solvent^c^	120	60	30	99
35	No catalyst	0	MeOH	120	60	1	99

The data reveals the catalysts’ optimal conditions and efficiencies.

Reaction conditions: Each reaction utilized 1 mmol of styrene oxide and 1 mmol of methanol. Conversion and selectivity data were obtained via gas chromatography.

^a^ $${\text{Mol\% of catalyst}} = \left( {\text{mol of catalyst}} \right)/\left( {\text{mol of epoxide}} \right) \times 100$$.

^b^In reactions where methanol served both as the nucleophile and the solvent, an excess amount of 2 ml of methanol was employed.

^c^Only 1 mmol of methanol was used as nucleophile.

Different solvents including toluene, tetrahydrofuran, dichloromethane, n-hexane, water, acetonitrile, ethyl acetate, and methanol were tested (Table 1). Methanol, doubling as the alcoholic nucleophile and solvent, yielded the best results. The reaction time was optimized by monitoring the conversion rates at various intervals, and 25 min was identified as the optimal duration for the styrene oxide and methanol reaction at room temperature. Therefore, 0.3 mol% of MIP catalyst, 0.5 ml of methanol at ambient temperature for 25 min, were determined as the ideal reaction conditions. Subsequently, the effect of different parameters on the reaction using UiO-66-(CO_2_H)_2_ catalyst was investigated. An increase in the catalyst amount led to higher conversion, with 0.9 mol% being sufficient for successful reaction progression. Unlike MIP, UiO required a higher temperature for catalysis. Increasing the temperature from room temperature to 60 °C for 2 h significantly improved the conversion rate from 65 to 98%. Upon varying the reaction time while keeping other parameters constant, the most effective duration for UiO was identified as 2 h, four times longer than required for MIP. Again, methanol emerged as the best solvent choice for UiO, both as a solvent and a nucleophile. Ultimately, 0.9 mol% of UiO catalyst for 120 min in methanol was found optimal for the model reaction. Comparative analysis revealed that MIP was more efficient than UiO in terms of catalyst amount, temperature, and time for the styrene oxide ring-opening reaction. Finally, to assess the performance of MIP-a, the ring opening of styrene oxide with methanol was conducted under the optimal conditions established for MIP. The results indicated that MIP-a is less effective in catalyzing the ring opening of styrene oxide under the same conditions compared to MIP (Table 1 entries 5 and 6). This is likely due to the broader PXRD pattern of MIP, which indicate more structural defects and greater accessibility of active catalytic sites for the reaction. Literatures support the fact that structural defects in MOFs can drastically improve catalytic activity^40^. Zhao et al., demonstrated that increased structural defects in Zr-UiO-66 MOF is in line with higher catalytic activity in the ring-opening of epoxides with alcohols. Defects in MOF structures act as active sites that facilitate chemical reactions, creating additional active sites and improving reaction efficiency. Defect-rich Zr-UiO-66 MOFs exhibited broader PXRD patterns, indicating lower crystallinity and higher defect density, which improves catalytic performance^23^. On the other hand, Liu et al. found that MOFs with higher defect densities show improved catalytic activity in epoxide ring-opening reactions, confirming a quantitative relationship between the number of defects and catalytic activity^41^. This concept can be used to explain the superior performance of the MIP catalyst. The presence of structural defects in MIP provides more active zirconium sites that are not coordinated by the aspartic acid linkers, improving the catalyst’s ability to facilitate ring-opening reactions. Furthermore, the enhanced proton transfer ability of MIP, allows for more protons transfer per unit area in less time compared to UiO, which in turn contributes to its higher catalytic activity. This synergistic effect of the metal centers and aspartic acid linkers in MIP results in a more efficient catalytic process, lowering the required catalyst amount, reaction time, and temperature.

**Figure 4:**
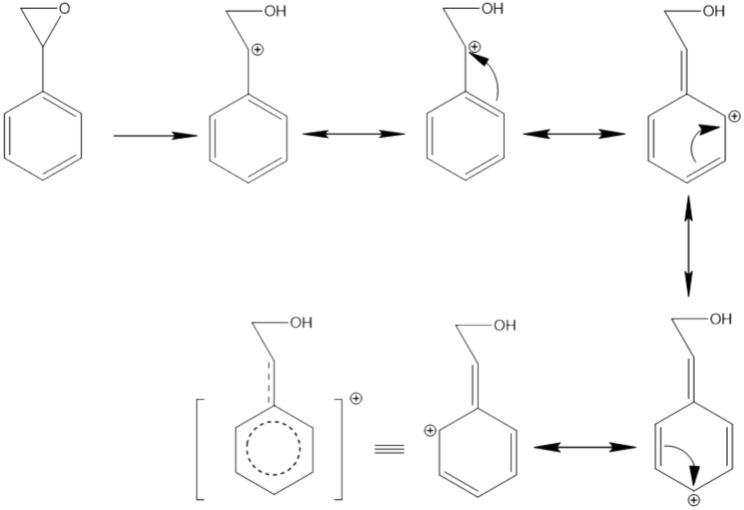
Delocalization of positive charge in styrene oxide.

After establishing optimal conditions for the ring-opening of epoxides by alcohols in the presence of MIP and UiO catalysts, a wide array of alcohols and epoxides was utilized to assess the performance of these catalysts under various conditions. Drawing upon the data presented in Table 2, the subsequent analysis of these results indicates a significant trend in reaction kinetics. When the nucleophile was changed from methanol to propanol, which possesses a longer carbon chain and greater steric hindrance, a noticeable reduction in the reaction rate was observed. This change is a clear indication of how molecular size and structure can significantly influence the efficiency of the reaction. Moreover, the benzyl group in styrene oxide is able to delocalize the positive charge formed during the ring-opening step (Fig. 4)^42^. This makes the benzylic carbon more electrophilic and susceptible to attack by the nucleophile. In contrast, the positive charge cannot be stabilized effectively for alkyl epoxides like cyclohexene oxide. Hence, ring-opening is slower for alkyl epoxides.

**Table 2:** Comparative catalytic performance of MIP and UiO in epoxide ring-opening reactions with various alcoholic nucleophiles.

Entry	Catalyst	Epoxide	Alcohols (Nu)	Major product	Time	Conversion	Selectivity
1	MIP		CH_3_OH		25 min	99	99
2	UiO		CH_3_OH		2 h	99	99
3	MIP		CH_3_CH_2_OH		10 h	99	99
4	UiO		CH_3_CH_2_OH		8 h	98	99
5	MIP		CH_3_CH_2_CH_2_OH		24 h	30	99
6	UiO		CH_3_CH_2_CH_2_OH		24 h	45	99
7	MIP		CH_3_OH		8 h	99	99
8	UiO		CH_3_OH		8 h	99	99
9	MIP		CH_3_CH_2_OH		18 h	99	99
10	UiO		CH_3_CH_2_OH		18 h	97	99
11	MIP		CH_3_CH_2_CH_2_OH		24 h	38	99
12	UiO		CH_3_CH_2_CH_2_OH		24 h	50	99
13	MIP		CH_3_OH		24 h	8	99
14	UiO		CH_3_OH		24 h	10	99
15	MIP		CH_3_CH_2_OH		24 h	3	99
16	UiO		CH_3_CH_2_OH		24 h	5	99
17	MIP		CH_3_CH_2_CH_2_OH		24 h	0	99
18	UiO		CH_3_CH_2_CH_2_OH		24 h	0	99

### Catalytic activity evaluation of the Zr-based MOFs in the epoxide ring-opening reactions by amine nucleophiles

To expand the understanding of the catalytic capabilities of MOFs beyond epoxide alcoholysis, we conduct an aminolysis case study. The reaction between styrene oxide and aniline was chosen as a model to optimize various parameters. This section discusses the influence of different parameters on the reaction of aniline with styrene oxide in the presence of UiO and MIP catalysts. As indicated in Table 3, a quantity of 0.92 mol% mg of UiO catalyst was found to be adequate for the reaction, slightly lower than the 1.02 mol% required for MIP. Notably, increasing the amount of UiO catalyst enhanced both selectivity and yield. In optimizing reaction time, while keeping other variables constant, it was observed that lengthening the reaction time from 1 to 2 h in the UiO catalyzed system increased the conversion rate from 70 to 98% at 60 °C (Table 3 entry 22). This duration is longer compared to the MIP-catalyzed reaction, which occurs efficiently at room temperature (Table 3 entry 3). Additionally, the impact of various solvents on the reaction is summarized in Table 3 (entries 6–9 and 25–28). It was found that both catalysts exhibit optimal catalytic activity in a solvent-free condition. For the UiO catalyst, a notable improvement was observed when the temperature was increased from 25 to 60 °C, with the best results achieved at 60 °C. In contrast, the MIP catalyst showed high efficiency at ambient temperature. Similar to the reactions with alcoholic nucleophiles, MIP outperformed UiO in terms of requiring less time and milder reaction conditions. The same factors contributing to the superior catalytic activity of MIP were also evident in this context. Finally, to evaluate the performance of MIP-a, the ring-opening reaction of styrene oxide with aniline was carried out under the optimal conditions established for MIP. The results demonstrated that MIP-a is less effective in catalyzing the ring-opening of styrene oxide under these conditions compared to MIP (Table 3 entries 4 and 5). The reasons mentioned in section “Catalytic activity evaluation of the Zr-based MOFs in the epoxide ring-opening reactions by alcohol nucleophiles” also apply here.

**Table 3:** A comparative evaluation of UiO and MIP catalysts highlights the impact of catalyst amount, temperature, time, and solvent on the styrene ring-opening reaction using amine nucleophile.


Entry	Catalyst	Catalyst amount (mol %)^a^	Solvent	Time (min)	Temperature (°C)	Conversion (%)	Selectivity (%)
1	MIP	0.34	Solvent free	120	r.t	80	90
2	MIP	0.68	Solvent free	120	r.t	90	90
3	MIP	1.02	Solvent free	60	r.t	95	93
4	MIP	1.02	Solvent free	120	r.t	97	93
5	MIP-a	1.02	Solvent free	120	r.t	63	93
6	MIP	1.02	THF	120	r.t	15	93
7	MIP	1.02	Toluene	120	r.t	40	93
8	MIP	1.02	CH_3_CN	120	r.t	30	90
9	MIP	1.02	DCM	120	r.t	18	90
10	No catalyst	0	Solvent free	120	r.t	5	90
11	UiO	0.23	Solvent free	120	r.t	43	90
12	UiO	0.46	Solvent free	120	r.t	48	90
13	UiO	0.69	Solvent free	120	r.t	52	90
14	UiO	0.92	Solvent free	120	r.t	60	90
15	UiO	0.92	Solvent free	120	40	65	90
16	UiO	0.92	Solvent free	120	50	72	93
17	UiO	0.23	Solvent free	120	60	67	90
18	UiO	0.46	Solvent free	120	60	84	90
19	UiO	0.69	Solvent free	120	60	95	93
20	UiO	0.92	Solvent free	60	60	70	90
21	UiO	0.92	Solvent free	90	60	88	93
22	UiO	0.92	Solvent free	120	60	98	93
23	UiO	1.15	Solvent free	120	60	98	93
24	No catalyst	0	Solvent free	120	60	20	90
25	UiO	0.92	THF	120	60	20	87
26	UiO	0.92	Toluene	120	60	42	93
27	UiO	0.92	CH_3_CN	120	60	37	42
28	UiO	0.92	DCM	120	60	15	90

Reaction conditions: Each reaction utilized 1 mmol of styrene oxide and 1 mmol of aniline. Conversion and selectivity data were obtained via gas chromatography.

^a^ $${\text{Mol\% of catalyst}} = \left( {\text{mol of catalyst}} \right)/\left( {\text{mol of epoxide}} \right) \times 100.$$

To evaluate the strengths of the catalysts, some aniline-based nucleophiles were used to investigate the ring-opening of styrene oxide under optimized conditions (Table 4). According to the results in Table 4, aniline attacked the less sterically hindered carbon of the epoxide ring, leading to a relatively fast reaction and good yield. The electron-withdrawing nitro substituents in 2-nitroaniline and 4-nitroaniline reduce the nucleophilicity of the amine and diminish both reaction rate and yield. The para-nitro group in 4-nitroaniline withdraws electrons more effectively through resonance stabilization compared to the ortho-nitro group in 2-nitroaniline. This stronger electron-withdrawing effect accounts for the slower reaction and lower yield with 4-nitroaniline. While the ortho-nitro group in 2-nitroaniline causes some steric encumbrance, the para position of the nitro group in 4-nitroaniline has less steric effects but more potent electron withdrawal based on the results in Table 4. In contrast, the electron-donating alkyl substituent in 4-n-butylaniline increases the electron density on nitrogen, improving its nucleophilicity. However, the butyl group also introduces steric congestion which decreases the reaction rate, though the electron-donating effect still enables reasonable yields under the specified reaction conditions.

**Table 4:** Comparative evaluation of MIP and UiO catalysts in epoxide ring-opening reactions with various amine nucleophiles.

Entry	Catalyst	Epoxide	Amine	Major product	Time (h)	Conversion (%)	Selectivity (%)
1	MIP				1	95	93
2	UiO				2	97	93
3	MIP				2	93	82
4	UiO				2	98	82
5	MIP				2	94	80
6	UiO				2	98	80
7	MIP				2.5	93	90
8	UiO				2.5	98	90

As illustrated in Table 5, an evaluation of the efficiency of MIP-202(Zr) and UiO-66-(CO_2_H)_2_ compared to existing catalysts in the ring opening of styrene oxide using methanol and aniline nucleophile is summarized. MIP exhibits an outstanding catalytic activity for the ring-opening of styrene oxide compared to the reported catalysts in literature which require substantially higher temperatures (40–60 °C), prolonged reaction times, and increased catalyst quantities to reach comparable conversion levels. MIP also demonstrates higher turnover frequencies than other catalysts when methanol or aniline are employed as nucleophiles. In addition to its superior performance, MIP possesses suitable biocompatibility and is economically viable. Moreover, this catalyst has the capability for scale-up and industrialization, which distinguishes it from the other examined catalysts.

**Table 5:** Comparative performance analysis of various catalysts in the ring-opening reaction of styrene oxide.

Catalyst	Nucleophile	Time (min)	Temperature (°C)	Yield (%)	TOF (h^−1^)^a^	References
MIL-pip-SO_3_H	Methanol	20 min	r.t	99.4	91.1	^32^
MIL-DABCO-SO_3_H	Methanol	30 min	r.t	98.3	89.4	^32^
MIL-101(HPW)	Methanol	20 min	40	99	98.5	^43^
Cu-MOF	Methanol	120 min	r.t	90	5.3	^44^
Fe-BTC	Methanol	60 min	40	83	10	^45^
HKUST-1	Methanol	150 min	40	94	5.6	^46^
PNVP/TiCl_4_	Methanol	15 min	r.t	98	80	^47^
MIL-101(Cr)-Pyz-RSO_3_H	Methanol	20 min	r.t	99	94	^42^
MIP	Methanol	25 min	r.t	99	707	This study
MIP-a	Methanol	25 min	r.t	70	499	This study
UiO-66(CO_2_H)_2_	Methanol	120 min	60	99	54	This study
Zr-BDC-MOF	Aniline	10 min	50	99.4	41	^48^
Zn-BTC-MOF	Aniline	10 min	50	98.3	21	^48^
Cu-BTC-MOF	Aniline	10 min	50	99	63	^48^
AlCl_3_	Aniline	120 min	60	98.7	49.6	^28^
Al(Otf)_3_	Aniline	120 min	60	82.5	43.65	^28^
Sn(OTf)_2_	Aniline	120 min	60	90.4	46.55	^28^
[tespmim][OTf]-Al(OTf)_3_	Aniline	120 min	60	99.9	49.95	^28^
CaO-SiO_2_-IL	Aniline	120 min	60	51.13	37	^28^
MIP	Aniline	60 min	r.t	99	107	This study
MIP-a	Aniline	60 min	r.t	62	67	This study
UiO-66-(CO_2_H)_2_	Aniline	120 min	60	99	55.5	This study

^a^ $${\text{TOF}}\left( {{\text{h}}^{ - 1} } \right) = { }\frac{{\text{mmol of converted styrene oxide}}}{{{\text{mmol of catalyst }} \times {\text{ Time of reaction }}\left( {\text{ hour}} \right)}}$$.

Based on the results obtained, it is evident that the MIP catalyst demonstrates significantly better performance in the ring-opening of epoxides by both alcoholic and amine nucleophiles compared to MIP-a and UiO. Several factors contribute to the superior catalytic activity of MIP. The comparison of the PXRD patterns of the catalysts shows that MIP has a broader PXRD pattern, indicating lower crystallinity compared to MIP-a and UiO. This broadening suggests a higher number of uncoordinated zirconium sites due to the presence of structural defects. These defects enhance the accessibility of active catalytic sites, thereby improving the overall catalytic performance. Additionally, in comparison with UiO, MIP not only has more structural defects but also exhibits higher proton conductivity, which contributes to its superior catalytic activity.

### Possible catalytic cycle for nucleophilic ring-opening reactions by Zr-based MOFs

**Figure 5:**
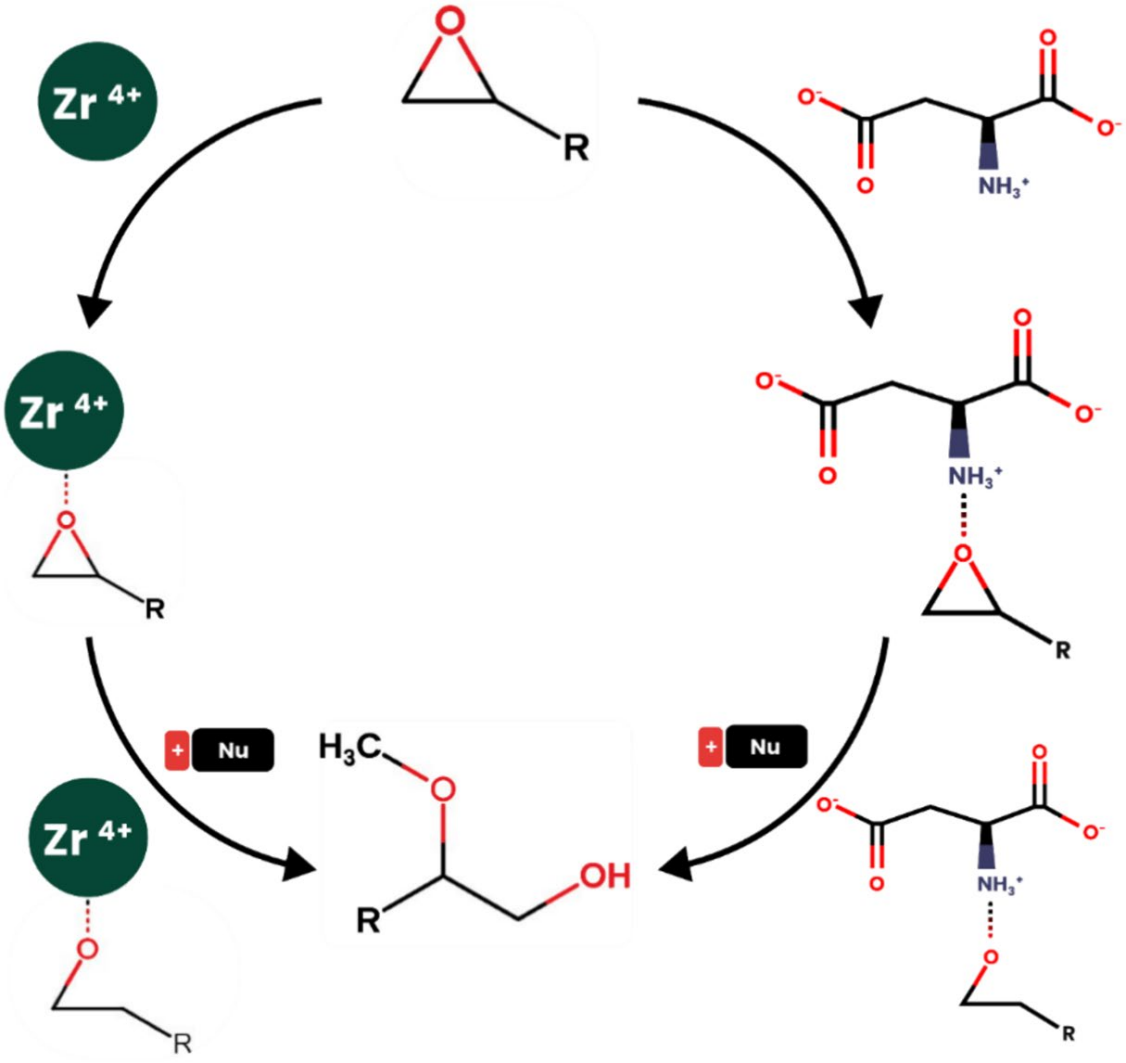
Possible catalytic cycle for nucleophilic ring-opening reaction by Zr-based MOFs.

Previous studies indicate that materials with acidic properties can accelerate epoxide ring-opening reactions. These materials can exhibit Lewis acidity, such as the metal centers in zirconium, copper, or iron-based metal–organic frameworks (MOFs) acting as Lewis acids^25^^49–53^, or Brønsted acidity, like the linkers in reported MOFs such as –SO_3_H and other similar compounds^33,42,54–57^. Therefore, in metal–organic frameworks, both the metal centers and linkers can facilitate epoxide ring-opening reactions if they possess acidic properties. In this study zirconium metal centers serve as active catalytic sites, while the acidic linkers assist in advancing the reaction (Fig. 5). According to Wang et al.^29^, who first introduced MIP-202, the aspartic acid linker in MIP-202 connects to zirconium clusters through its carboxylate groups, leaving the NH_3_^+^ group available. This NH_3_^+^ group functions as a hydrogen bond donor, interacting with the oxygen atom in epoxides and thereby facilitating the C–O bond cleavage. In the case of UiO-66-(CO_2_H)_2_, the linker 1,2,4,5-benzenetetracarboxylic acid connects to zirconium clusters via two of its four carboxylate groups, with the remaining two carboxylic acid groups also participating in the reaction by interacting with the epoxides.

To further confirm the catalytic mechanism of MIP, we employed a masking experiment similar to the method used by Das et al.^25^. Pyridine, a known masking agent, was introduced to the reaction mixture. ZrCl_4_, previously identified as an effective catalyst for epoxide ring-opening reactions, was tested, and its catalytic activity was found to be completely inhibited by pyridine. This inhibition indicates that the zirconium metal centers in ZrCl_4_ and MIP-202 are crucial active sites. We then compared the activity of our zirconium-based MIP with its corresponding metal salt. As illustrated in Figure S7, catalytic activity of MIP is significantly higher than that of ZrCl_4_ (the metal component). This enhanced activity is attributed to the dual active sites present in the MOFs: the zirconium metal nodes (Lewis acidic sites) and the aspartic acid linkers (Brønsted acidic sites). The introduction of pyridine to the reaction mixture resulted in a complete quenching of the MOF's catalytic activity. This observation strongly suggests that pyridine acts as a Lewis base, binding firmly to the metal ion and preventing the substrate from interacting with the metal centers. Additionally, the catalytic activity of MIP was quenched due to the formation of hydrogen bonds between the NH_3_^+^ groups of the aspartic acid linker and the nitrogen atom of pyridine. These findings confirm the crucial role of both the metal centers and the acidic linkers in facilitating the epoxide ring-opening reaction.

### Reusability

**Figure 6:**
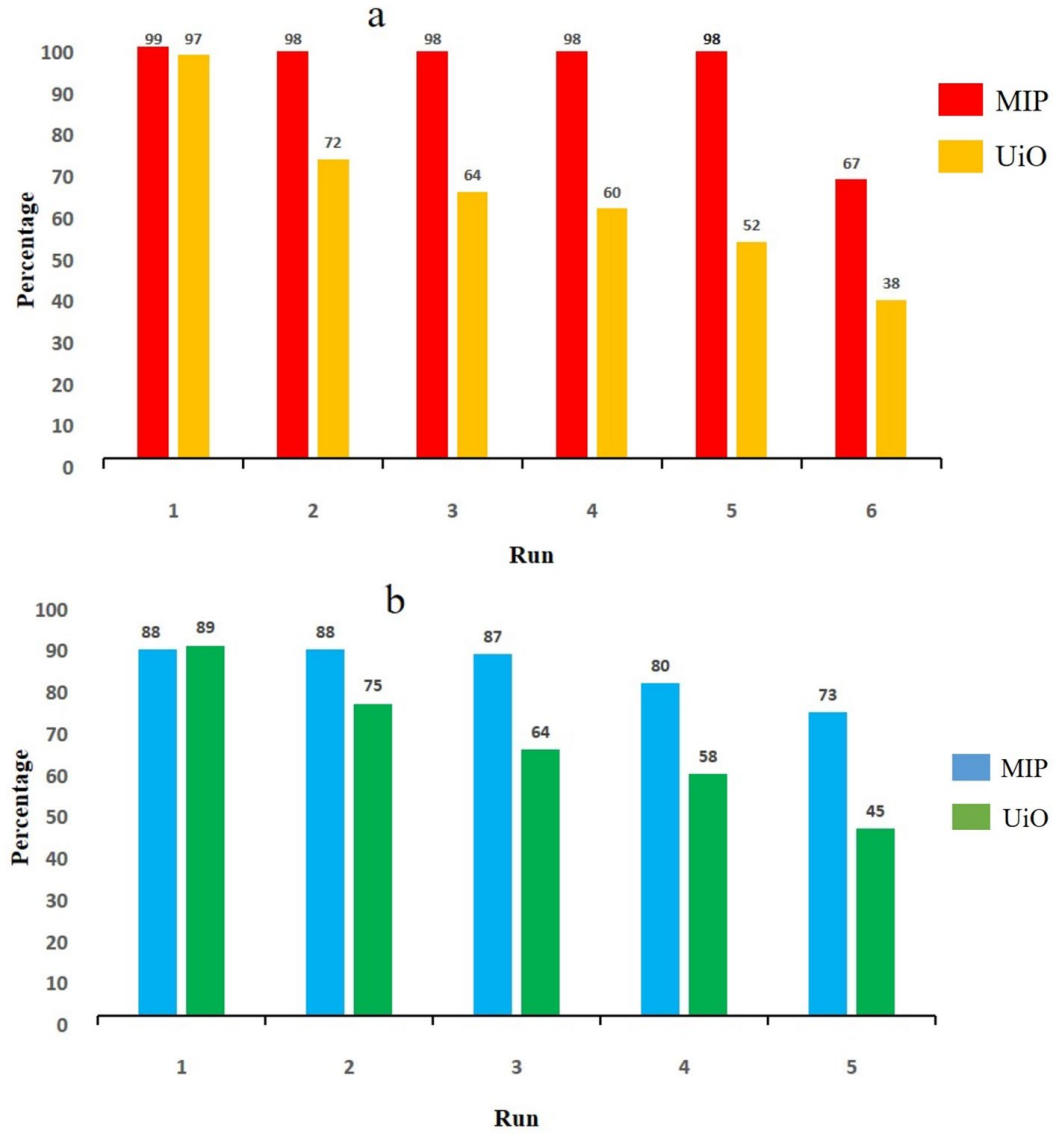
Reusability of MIP-202(Zr) and UiO-66-(CO_2_H)_2_ in the ring-opening of styrene oxide using (**a**) methanol and (**b**) aniline.

Using the model reaction of styrene oxide ring-opening by methanol and aniline, we evaluated the catalytic performance of MIP and UiO over consecutive reaction cycles under consistent conditions. MIP demonstrated exceptional stability and reusability, maintaining its high efficiency and selectivity over five repeated uses for methanol and three cycles for aniline without significant degradation. In contrast, UiO exhibited steadily declining activity after initial use in both ring-opening reactions (Fig. 6).

**Figure 7:**
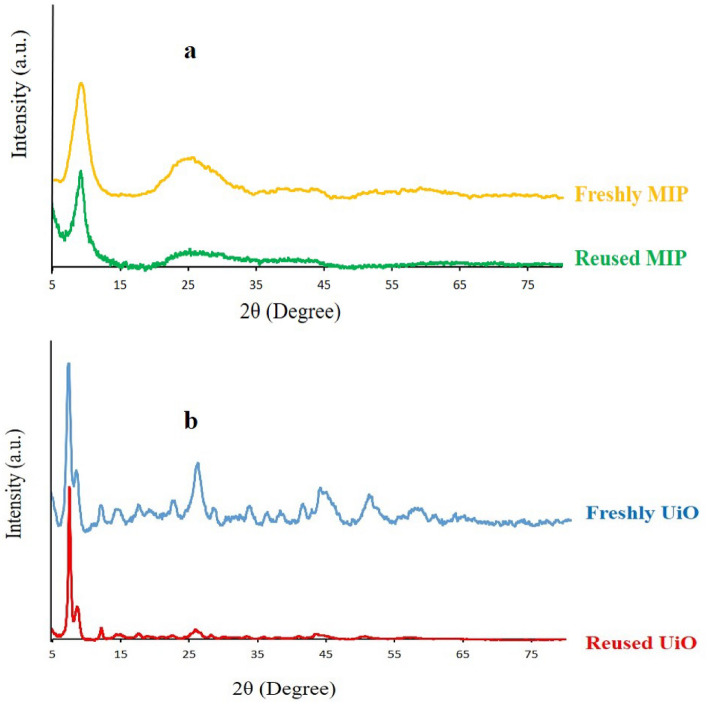
The PXRD patterns of freshly prepared vs. reused MOFs in the ring-opening of styrene oxide using methanol: (**a**) MIP, (**b**) UiO.

To further investigate their stability, we performed PXRD analysis for both MIP and UiO after catalytic reactions (Fig. 7). The PXRD patterns show only minor changes, indicating that both materials maintain their structural integrity after catalytic use. It seems the harsher reaction conditions required for UiO catalyst, including higher temperatures and longer reaction times, likely contribute to catalyst poisoning and pore blockage by reactants, leading to a higher likelihood of active site contamination and faster catalytic activity decline. In contrast, MIP-202(Zr) operates under milder conditions, which helps to minimize these issues and maintain its stability. Moreover, MIP-202(Zr) contains a higher number of structural defects, resulting in a greater number of active catalytic sites compared to UiO-66-(CO_2_H)_2_. These structural defects enhance mass transport and create additional active sites, thereby sustaining its catalytic performance over more cycles. As mentioned above literature supports the notion that increased structural defects in MOFs can significantly boost catalytic activity by providing more accessible active sites. These factors collectively contribute to the superior catalytic stability and reusability of MIP compared to UiO.

### Leaching test

**Figure 8:**
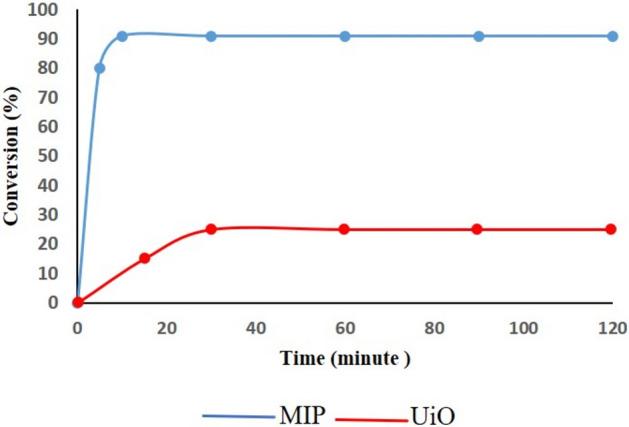
Leaching test of MIP and UiO.

To evaluate the leaching behavior of the catalysts, leaching tests were performed for both MIP and UiO under optimized reaction conditions. For MIP, the reaction between styrene oxide and methanol was conducted, and after 10 min, the catalyst was separated by centrifugation. The reaction continued without the catalyst for an additional 110 min, and conversion was monitored using GC. The results showed that the conversion stabilized at 91%, with no further conversion observed, indicating that the catalyst did not leach into the solution. Similarly, for UiO, the reaction between styrene oxide and methanol was conducted at 60 °C, and after 30 min, the catalyst was separated by centrifugation. The reaction continued without the catalyst for an additional 1.5 h, and conversion was monitored using GC. The results showed that the conversion initially stabilized at 20% for UiO. However, no further increase in conversion was observed over the subsequent 90 min, indicating that UiO does not leach into the reaction solution (Fig. 8). These results confirm that both MIP and UiO act as true heterogeneous catalysts without leaching into the reaction solution.

## Conclusion

This study has successfully demonstrated the superior catalytic performance of the Zr-based metal–organic framework (MOF) MIP-202(Zr) in the ring-opening reactions of epoxides using both alcohols and amines as nucleophiles. Through a detailed comparative analysis with UiO-66-(CO_2_H)_2_, several key findings have emerged: MIP-202(Zr) exhibits remarkable catalytic efficiency under significantly milder conditions compared to UiO-66-(CO_2_H)_2_. For instance, MIP-202(Zr) achieved 99% conversion of styrene oxide in 25 min at room temperature with methanol as the nucleophile, whereas UiO-66-(CO_2_H)_2_ required 120 min at 60 °C with four times the catalyst loading to reach a similar conversion. Similar trends were observed in reactions with aniline, where MIP-202(Zr) showed higher efficiency and faster reaction rates. The superior performance of MIP-202(Zr) can be attributed to its cooperative Brønsted/Lewis acid sites and higher proton conductivity, which enable more efficient epoxide activation. Additionally, the presence of structural defects in MIP-202(Zr) enhances its catalytic activity by providing more accessible active sites. The study also confirmed the exceptional stability and reusability of MIP-202(Zr) over multiple cycles, maintaining high activity and selectivity, whereas UiO-66-(CO_2_H)_2_ showed a gradual decline in performance. Overall, the findings highlight the potential of MIP-202(Zr) as a cost-effective and sustainable catalyst for industrial-scale applications in diverse epoxide ring-opening reactions.

### Supplementary Information

Supplementary Information.

## Data Availability

The data that support the findings of this study, including starting materials, NMR spectra of products, GC data for model reactions, and analytical methods employed, are available at the supporting information file.

## References

[1] Fałtynowicz, H., Kułażynski, M. & Goodman, S. H. Epoxies. *Handb. Thermoset Plast.* **2002**, 175–229. 10.1016/B978-0-12-821632-3.00014-2 (2022). 10.1016/B978-0-12-821632-3.00014-2

[2] Thirumalaikumar, M. Ring opening reactions of epoxides: A review. *Org. Prep. Proced. Int.* **54**, 1–39 (2022). 10.1080/00304948.2021.1979357

[3] Moser, B. R., Cermak, S. C., Doll, K. M., Kenar, J. A. & Sharma, B. K. A review of fatty epoxide ring opening reactions: Chemistry, recent advances, and applications. *J. Am. Oil Chem. Soc.* **99**, 801–842 (2022). 10.1002/aocs.12623

[4] Jacobsen, E. N. Asymmetric catalysis of epoxide ring-opening reactions. *ChemInform* **2000**, 31 (2000). 10.1021/ar960061v 10891060

[5] Moschona, F., Savvopoulou, I., Tsitopoulou, M., Tataraki, D. & Rassias, G. Epoxide syntheses and ring-opening reactions in drug development. *Catalysts* **10**, 1–65 (2020). 10.3390/catal10101117

[6] Tyagi, A., Yadav, N., Khan, J., Mondal, S. & Hazra, C. K. Brønsted acid-catalysed epoxide ring-opening using amine nucleophiles: A facile access to β-amino alcohols. *Chem. Asian J.* **2022**, 17 (2022). 10.1002/asia.202200379 35485456

[7] Deshpande, N. *et al.* Epoxide ring opening with alcohols using heterogeneous Lewis acid catalysts: Regioselectivity and mechanism. *J. Catal.* **370**, 46–54 (2019). 10.1016/j.jcat.2018.11.038

[8] Bruno, S. M. *et al.* Catalytic alcoholysis of epoxides using metal-free cucurbituril-based solids. *Org. Biomol. Chem.* **14**, 3873–3877 (2016). 27035403 10.1039/C6OB00512H

[9] Barluenga, J., Vázquez-Villa, H., Ballesteros, A. & González, J. M. Copper(II) tetrafluoroborate catalyzed ring-opening reaction of epoxides with alcohols at room temperature. *Org. Lett.* **4**, 2817–2819 (2002). 12182563 10.1021/ol025997k

[10] Li, D. *et al.* Highly regioselective ring-opening of epoxides with amines: A metal—and solvent-free protocol for the synthesis of β-amino alcohols. *Chem. Commun.* **56**, 2256–2259 (2020). 10.1039/C9CC09048G 31984384

[11] Brunelli, N. A., Long, W., Venkatasubbaiah, K. & Jones, C. W. Catalytic regioselective epoxide ring opening with phenol using homogeneous and supported analogues of dimethylaminopyridine. *Top. Catal.* **55**, 432–438 (2012). 10.1007/s11244-012-9822-2

[12] Grubbs, R. H. Olefin-metathesis catalysts for the preparation of molecules and materials (nobel lecture). *Angew. Chem. Int. Ed.* **45**, 3760–3765 (2006). 10.1002/anie.200600680 16724297

[13] Stahl, S. S. Organotransition metal chemistry: From bonding to catalysis. *J. Am. Chem. Soc.* **132**, 8524–8525 (2010). 10.1021/ja103695e

[14] Bavykina, A. *et al.* Metal-organic frameworks in heterogeneous catalysis: Recent progress, new trends, and future perspectives. *Chem. Rev.* **120**, 8468–8535 (2020). 32223183 10.1021/acs.chemrev.9b00685

[15] Grefe, L. & Mejía, E. Earth-abundant bimetallic and multimetallic catalysts for Epoxide/CO_2_ ring-opening copolymerization. *Tetrahedron* **98**, 132433 (2021). 10.1016/j.tet.2021.132433

[16] Yadav, R. *et al.* Aluminium oxide supported on SBA-15 molecular sieves as potential lewis acid catalysts for epoxide ring opening using aniline. *Catal. Lett.* **148**, 1407–1415 (2018). 10.1007/s10562-018-2366-8

[17] Chakraborti, A. K., Rudrawar, S. & Kondaskar, A. An efficient synthesis of 2-amino alcohols by silica gel catalysed opening of epoxide rings by amines. *Org. Biomol. Chem.* **2**, 1277–1280 (2004). 15105916 10.1039/b400588k

[18] Furukawa, H., Cordova, K. E., O’Keeffe, M. & Yaghi, O. M. The chemistry and applications of metal-organic frameworks. *Science.* **2013**, 341 (2013). 10.1126/science.1230444 23990564

[19] Sabo, M., Henschel, A., Fröde, H., Klemm, E. & Kaskel, S. Solution infiltration of palladium into MOF-5: Synthesis, physisorption and catalytic properties. *J. Mater. Chem.* **17**, 3827–3832 (2007). 10.1039/b706432b

[20] Hendon, C. H. & Walsh, A. Chemical principles underpinning the performance of the metal-organic framework HKUST-1. *Chem. Sci.* **6**, 3674–3683 (2015). 28706713 10.1039/C5SC01489A PMC5496192

[21] Shin, J. H. *et al.* Solubility selectivity-enhanced SIFSIX-3-Ni-containing mixed matrix membranes for improved CO_2_/CH_4_ separation efficiency. *J. Memb. Sci.* **633**, 119390 (2021). 10.1016/j.memsci.2021.119390

[22] Bai, Y. *et al.* Zr-based metal–organic frameworks: Design, synthesis, structure, and applications. *Chem. Soc. Rev.* **45**, 2327–2367 (2016). 26886869 10.1039/C5CS00837A

[23] Zhao, H. *et al.* Solid-state synthesis of defect-rich Zr-UiO-66 metal-organic framework nanoparticles for the catalytic ring opening of epoxides with alcohols. *ACS Appl. Nano Mater.* **4**, 9752–9759 (2021). 10.1021/acsanm.1c02156

[24] Jrad, A., Damacet, P., Yaghi, Z., Ahmad, M. & Hmadeh, M. Zr-based metal-organic framework nanocrystals for water remediation. *ACS Appl. Nano Mater.* **5**, 10795–10808 (2022). 10.1021/acsanm.2c02128

[25] Das, A., Anbu, N., Reinsch, H., Dhakshinamoorthy, A. & Biswas, S. A Thiophene-2-carboxamide-functionalized Zr(IV) organic framework as a prolific and recyclable heterogeneous catalyst for regioselective ring opening of epoxides. *Inorg. Chem.* **58**, 16581–16591 (2019). 31750650 10.1021/acs.inorgchem.9b02608

[26] Huang, Z. Q. *et al.* A novel copper framework with amino tridentate N-donor ligand as heterogeneous catalyst for ring opening of epoxides. *Appl. Organomet. Chem.* **35**, e6262 (2021). 10.1002/aoc.6262

[27] Olia, F. K., Sayyahi, S. & Taheri, N. An Fe_3_O_4_ nanoparticle-supported Mn (II)-azo Schiff complex acts as a heterogeneous catalyst in alcoholysis of epoxides. *Compt. Rend. Chim.* **20**, 370–376 (2017). 10.1016/j.crci.2016.06.007

[28] Latos, P. *et al.* Highly stable Lewis acidic trifloaluminate ionic liquid supported on silica and metallosilicates as an efficient catalyst for continuous flow aminolysis of epoxides. *Environ. Technol. Innov.* **31**, 103164 (2023). 10.1016/j.eti.2023.103164

[29] Wang, S. *et al.* A robust zirconium amino acid metal-organic framework for proton conduction. *Nat. Commun.* **9**, 1–8 (2018). 30467390 10.1038/s41467-018-07414-4 PMC6250719

[30] Yang, Y. & Xia, Y. Polycarboxyl metal–organic framework UiO-66-(COOH)_2_ as efficient desorption/ionization matrix of laser desorption/ionization mass spectrometry for selective enrichment and detection of phosphopeptides. *J. Nanoparticle Res.* **21**, 1–12 (2019). 10.1007/s11051-019-4646-7

[31] Tao, R. *et al.* Seeds-assisted synthesis of Zr-based metal-organic framework (MIP-202) for efficient adsorption separation of CO_2_/CH_4_ and CO_2_/N_2_. *J. Porous Mater.* **30**, 2129–2137 (2023). 10.1007/s10934-023-01494-4

[32] Mortazavi, S. S., Abbasi, A. & Masteri-Farahani, M. Influence of SO_3_H groups incorporated as Brønsted acidic parts by tandem post-synthetic functionalization on the catalytic behavior of MIL-101(Cr) MOF for methanolysis of styrene oxide. *Colloids Surfaces A Physicochem. Eng. Asp.* **599**, 124703 (2020). 10.1016/j.colsurfa.2020.124703

[33] Das, A., Anbu, N., Sk, M., Dhakshinamoorthy, A. & Biswas, S. Influence of hydrogen bond donating sites in UiO-66 metal-organic framework for highly regioselective methanolysis of epoxides. *ChemCatChem* **12**, 1789–1798 (2020). 10.1002/cctc.201902219

[34] Radojčić, D. *et al.* Study on the reaction of amines with internal epoxides. *Eur. J. Lipid Sci. Technol.* **118**, 1507–1511 (2016). 10.1002/ejlt.201500490

[35] Zhao, P. Q., Xu, L. W. & Xia, C. G. Transition metal-based lewis acid catalyzed ring opening of epoxides using amines under solvent-free conditions. *Synlett* **2004**, 846–850 (2004).

[36] Saddique, F. A. *et al.* Recent trends in ring opening of epoxides by amines as nucleophiles. *Synth. Commun.* **46**, 831–868 (2016). 10.1080/00397911.2016.1170148

[37] Diab, K. E., Salama, E., Hassan, H. S., Abd-El-moneim, A. & Elkady, M. F. Biocompatible MIP-202 Zr-MOF tunable sorbent for cost-effective decontamination of anionic and cationic pollutants from waste solutions. *Sci. Rep.* **11**, 1–13 (2021). 33758308 10.1038/s41598-021-86140-2 PMC7987968

[38] Wang, K., Wu, J., Zhu, M., Zheng, Y. Z. & Tao, X. Highly effective pH-universal removal of tetracycline hydrochloride antibiotics by UiO-66-(COOH)_2_/GO metal–organic framework composites. *J. Solid State Chem.* **284**, 121200 (2020). 10.1016/j.jssc.2020.121200

[39] Gao, Y., Pan, Y., Zhou, Z., Tian, Q. & Jiang, R. The carboxyl functionalized UiO-66-(COOH)_2_ for selective adsorption of Sr^2+^. *Molecules* **27**, 1208 (2022). 35208998 10.3390/molecules27041208 PMC8879016

[40] Dai, S. *et al.* Highly defective ultra-small tetravalent MOF nanocrystals. *Nat. Commun.* **15**, 1 (2024). 38653991 10.1038/s41467-024-47426-x PMC11039632

[41] Liu, Y., Klet, R. C., Hupp, J. T. & Farha, O. Probing the correlations between the defects in metal–organic frameworks and their catalytic activity by an epoxide ring-opening reaction. *Chem. Commun.* **52**, 7806–7809 (2016). 10.1039/C6CC03727E 27229848

[42] Mortazavi, S. S., Abbasi, A. & Masteri-Farahani, M. A new Brønsted acid MIL-101(Cr) catalyst by tandem post-functionalization; synthesis and its catalytic application. *Appl. Organomet. Chem.* **34**, e5717 (2020). 10.1002/aoc.5717

[43] Wee, L. H., Bonino, F., Lamberti, C., Bordiga, S. & Martens, J. A. Cr-MIL-101 encapsulated Keggin phosphotungstic acid as active nanomaterial for catalysing the alcoholysis of styrene oxide. *Green Chem.* **16**, 1351–1357 (2014). 10.1039/C3GC41988F

[44] Jiang, D., Mallat, T., Krumeich, F. & Baiker, A. Copper-based metal-organic framework for the facile ring-opening of epoxides. *J. Catal.* **257**, 390–395 (2008). 10.1016/j.jcat.2008.05.021

[45] Dhakshinamoorthy, A., Alvaro, M. & Garcia, H. Metal-organic frameworks as efficient heterogeneous catalysts for the regioselective ring opening of epoxides. *Chem. A Eur. J.* **16**, 8530–8536 (2010). 10.1002/chem.201000588 20549723

[46] Wee, L. H., Lohe, M. R., Janssens, N., Kaskel, S. & Martens, J. A. Fine tuning of the metal–organic framework Cu3(BTC)2 HKUST-1 crystal size in the 100 nm to 5 micron range. *J. Mater. Chem.* **22**, 13742–13746 (2012). 10.1039/c2jm31536j

[47] Rahmatpour, A. & Sajjadinezhad, S. M. Cross-linked poly(N-vinylpyrrolidone)-titanium tetrachloride complex: A novel stable solid TiCl_4_ equivalent as a recyclable polymeric Lewis acid catalyst for regioselective ring-opening alcoholysis of epoxides. *Appl. Organomet. Chem.* **35**, 1–17 (2021). 10.1002/aoc.6385

[48] Rani, P. & Srivastava, R. Nucleophilic addition of amines, alcohols, and thiophenol with epoxide/olefin using highly efficient zirconium metal organic framework heterogeneous catalyst. *RSC Adv.* **5**, 28270–28280 (2015). 10.1039/C5RA00921A

[49] Tanaka, K. *et al.* Asymmetric ring-opening reaction of meso -epoxides with aromatic amines using homochiral metal-organic frameworks as recyclable heterogeneous catalysts. *RSC Adv.* **8**, 28139–28146 (2018). 35542745 10.1039/C8RA05163A PMC9083937

[50] Julião, D. *et al.* Improved catalytic performance of porous metal–organic frameworks for the ring opening of styrene oxide. *CrystEngComm* **19**, 4219–4226 (2017). 10.1039/C7CE00528H

[51] Nagarjun, N., Concepcion, P. & Dhakshinamoorthy, A. MIL-101(Fe) as an active heterogeneous solid acid catalyst for the regioselective ring opening of epoxides by indoles. *Mol. Catal.* **482**, 110628 (2020). 10.1016/j.mcat.2019.110628

[52] Thornburg, N. E. *et al.* MOFs and their grafted analogues: Regioselective epoxide ring-opening with Zr6 nodes. *Catal. Sci. Technol.* **6**, 6480–6484 (2016). 10.1039/C6CY01093H

[53] Anbu, N. & Dhakshinamoorthy, A. Regioselective ring opening of styrene oxide by carbon nucleophiles catalyzed by metal–organic frameworks under solvent-free conditions. *J. Ind. Eng. Chem.* **58**, 9–17 (2018). 10.1016/j.jiec.2017.08.054

[54] Blandez, J. F. *et al.* Influence of functionalization of terephthalate linker on the catalytic activity of UiO-66 for epoxide ring opening. *J. Mol. Catal. A Chem.* **425**, 332–339 (2016). 10.1016/j.molcata.2016.10.022

[55] Gharib, M., Esrafili, L., Morsali, A. & Retailleau, P. Solvent-assisted ligand exchange (SALE) for the enhancement of epoxide ring-opening reaction catalysis based on three amide-functionalized metal–organic frameworks. *Dalt. Trans.* **48**, 8803–8814 (2019). 10.1039/C9DT00941H 31134242

[56] Mortazavi, S. S., Masteri-Farahani, M. & Abbasi, A. Ship-in-bottle preparation of multi-SO3H functionalized ionic liquid@MIL-100(Fe) for acid-catalyzed ring-opening of epoxides. *Appl. Organomet. Chem.* **35**, e6424 (2021). 10.1002/aoc.6424

[57] Mohammadikish, M., Valimohammadi, Z. & Masteri-Farahani, M. Post-synthetic modification of NH2-MIL88B(Fe) with sulfonic acid groups for acid-catalyzed epoxide ring-opening reaction. *CrystEngComm* **25**, 321–327 (2023). 10.1039/D2CE01289H

