# Supplementary material for: Superior and efficient performance of cost-effective MIP-202 catalyst over UiO-66-(CO2H)2 in epoxide ring opening reactions
Source: Sci Rep. 2024 Jul 31;14:17730. doi: 10.1038/s41598-024-68497-2 (PMC11291889; doi:10.1038/s41598-024-68497-2)
Supplement: Supplementary file 1 — Supplementary Information. [file 41598_2024_68497_MOESM1_ESM.docx]

Superior and efficient performance of cost-effective MIP-202 catalyst over UiO-66-(CO_2_H)_2_ in epoxide ring opening reactions

**Mojtaba Bagherzadeh**^a^**,** Mohsen Chegeni^a^, Arshad Bayrami^b^, Mojtaba Amini^c^

^a^Chemistry Department, Sharif University of Technology, Tehran, Iran

^b^Department of Chemistry, Research Center for Development of Advanced Technologies, Tehran, Iran

^c^Department of Inorganic Chemistry, Faculty of Chemistry, University of Tabriz, Tabriz, Iran

Corresponding authors; Mojtaba Bagherzadeh, Chemistry Department, Sharif University of Technology, PO Box 11155-3615, Tehran, Iran. Email: [bagherzadeh@sharif.edu](mailto:bagherzadeh@sharif.edu)

# 1. Materials and characterization techniques

## 1.1. **Material**

The compounds were obtained from Merck, Fluka, and Sigma-Aldrich chemical companies and were utilized as received without additional purification.

Table 1 S. List of analytically graded chemical reagents employed in the experimental methodologies.

| Chemical name | Chemical formula | Source of procurement |
| --- | --- | --- |
| Zirconium(IV) chloride | ZrCl_4_ | Sigma-Aldrich |
| L-Aspartic acid | C_4_H_7_NO_4_ | Sigma-Aldrich |
| Acetic acid | C_2_H_4_O_2_ | Merck |
| n-Hexane | C_6_H_14_ | Merck |
| Methanol | CH_4_O | Merck |
| Dichloromethane | CH_2_Cl_2_ | Merck |
| Ethanol | C_2_H_6_O | Merck |
| Tetrahydrofuran | C_4_H_8_O | Sigma-Aldrich |
| Toluene | C_7_H_8_ | Merck |
| Acetonitrile | C_2_H_3_N | Merck |
| Ethyl acetate | C_4_H_8_O_2_ | Merck |
| Styrene oxide | C_8_H_8_O | Fluka |
| Aniline | C_6_H_5_NH_2_ | Merck |
| 2-nitroaniline | C_6_H_6_N_2_O_2_ | Merck |
| 4-nitroaniline | C_6_H_6_N_2_O_2_ | Merck |
| 4-butylaniline | C_10_H_15_N | Merck |
| 7-oxabicyclo[4.1.0]heptane | C_6_H_10_O | Fluka |
| 9-oxabicyclo[6.1.0]nonane | C_8_H_14_O | Fluka |
| 1,2,4,5-Benzene tetracarboxylic acid | C_10_H_6_O_8_ | Merck |

## 1.2. Characterization techniques:

Nuclear Magnetic Resonance (NMR) spectra of compounds were recorded utilizing an Advanced Bruker-DRX (400MHz) instrument, with CDCl_3_ as the solvent. The chemical shifts were precisely measured and reported in parts per million (ppm), using tetramethylsilane (TMS) as the standard reference. The resonance magnetic multiplicity for hydrogen nuclei was categorized into various types, including singlet (s), doublet (d), triplet (t), quartet (q), multiplet (m), and broad (br), to provide detailed molecular insights. Carbon NMR spectra were also acquired using CDCl_3_ as the solvent, performed at 100MHz. The infrared (IR) spectra of the samples were acquired using an ABB Bomem MB-100 Fourier Transform Infrared (FTIR) Spectrometer. Samples were prepared as potassium bromide (KBr) pellets, and the spectra were meticulously recorded, with frequencies expressed in wavenumber units. Field Emission Scanning Electron Microscopy (FE-SEM) and Energy-Dispersive X-ray (EDX) analysis were conducted on an XMU-MIRA3 TESCAN device. These techniques were instrumental in determining the surface morphology, particle size, and elemental composition of the samples, providing a comprehensive understanding of their physical structure and chemical makeup. The products of the catalytic reactions were analyzed using Gas Chromatography (GC), performed on an Agilent Technologies 6890N system. This system was equipped with a 19091J-236 HP-5 5% phenyl methyl siloxane capillary column, ensuring accurate separation and identification of reaction components. X-ray Diffraction (XRD) patterns were obtained using an XPERT-PRO device from MPD Analytical Company, which employed a Cu Kα radiation source. XRD was crucial in determining the crystalline structure and phase composition of the materials, thereby playing a key role in understanding their catalytic behavior.

solvent


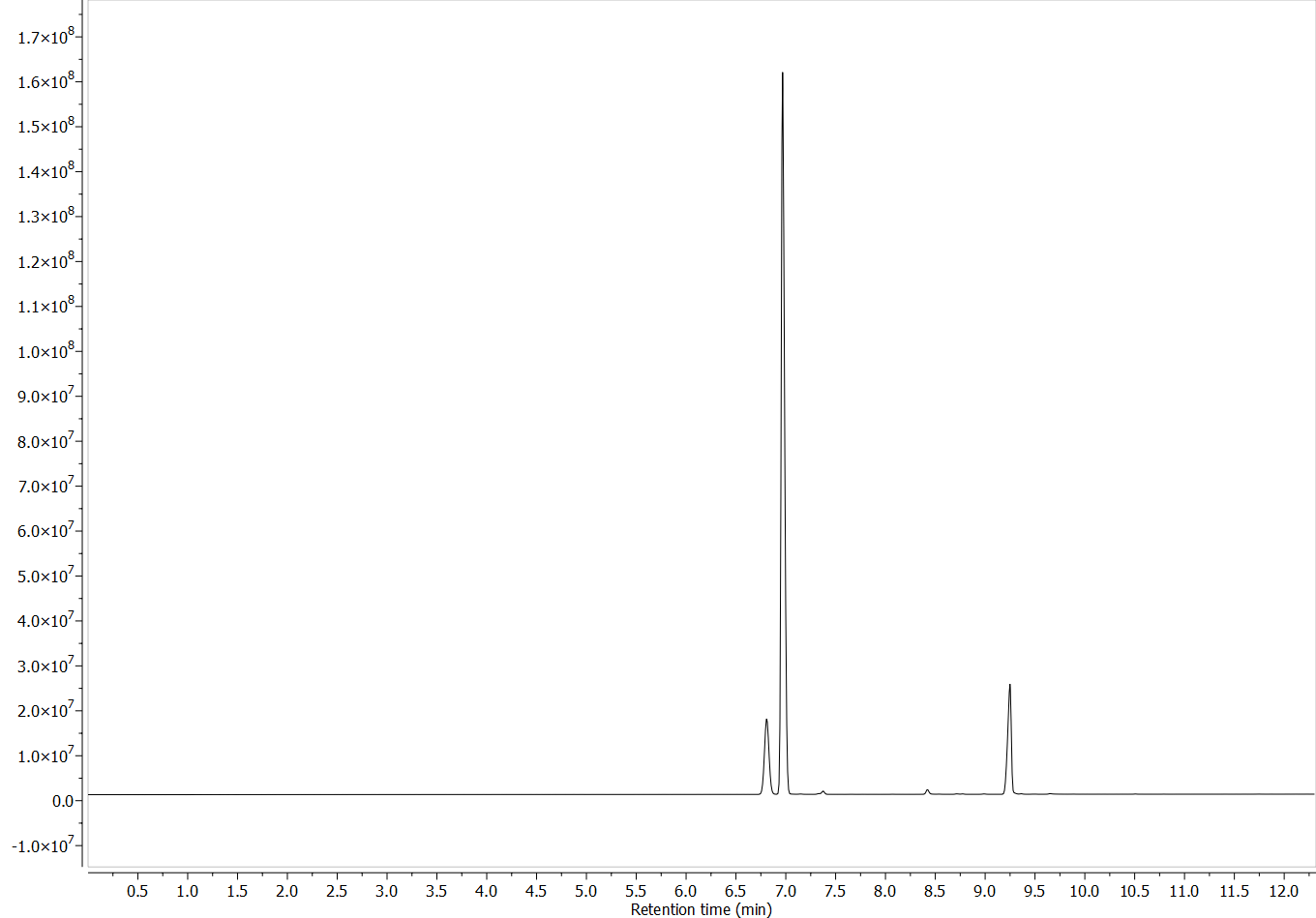


methanol

product

Figure S1. GC chromatogram of model reaction (ring opening of styrene oxide using methanol).


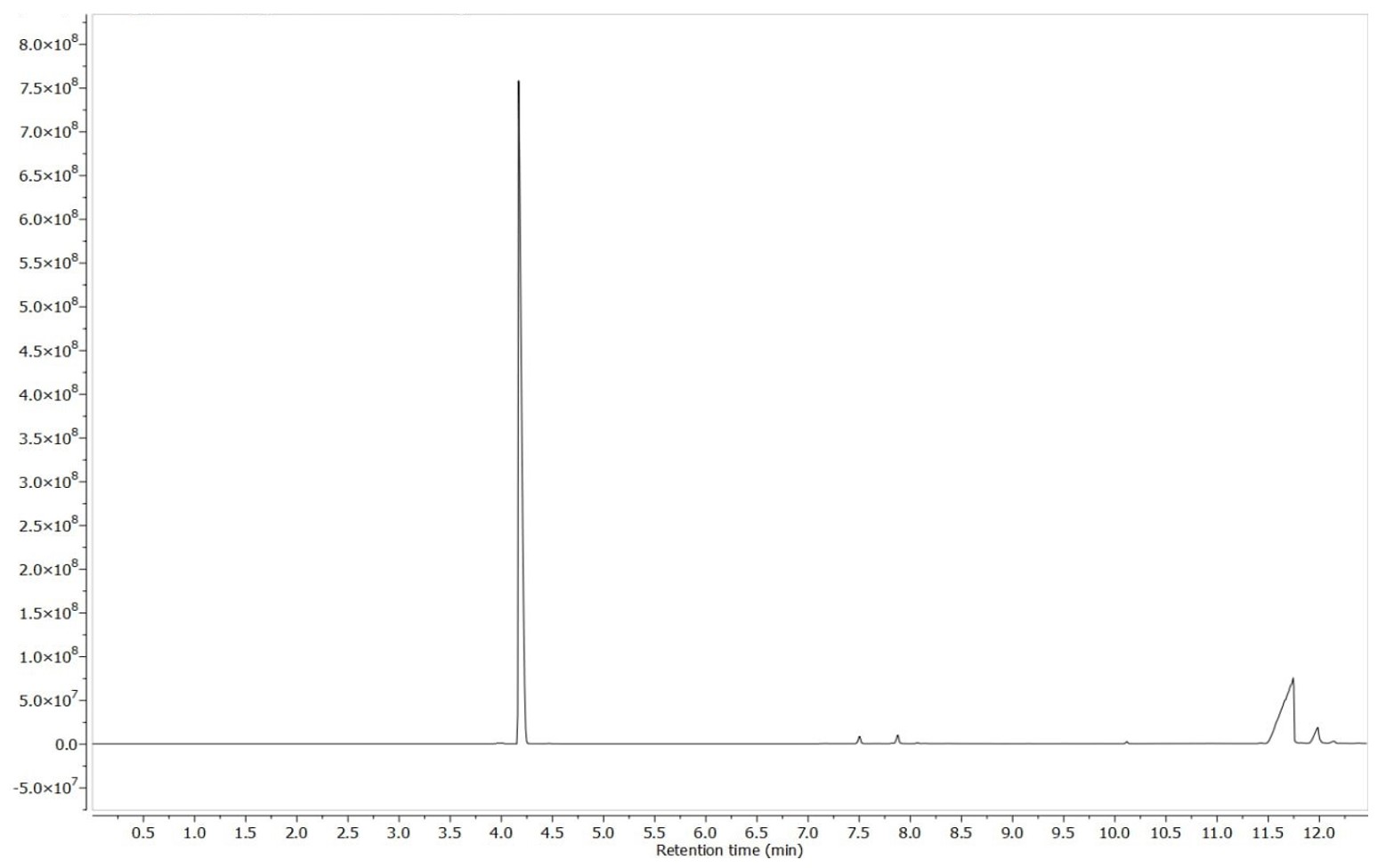


solvent

products

Figure S2. GC chromatogram of model reaction (ring opening of styrene oxide using aniline).


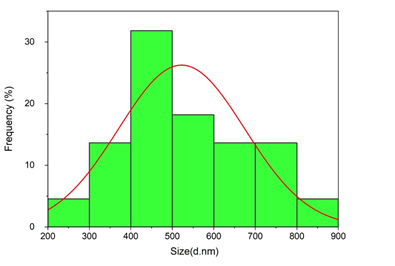


Figure S3. Size distribution of MIP.


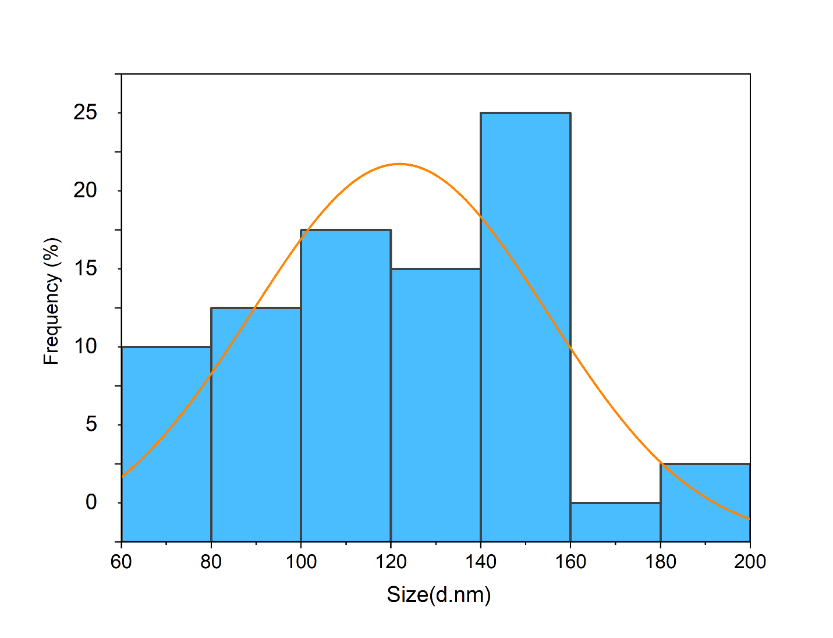


Figure S4. Size distribution of UiO.


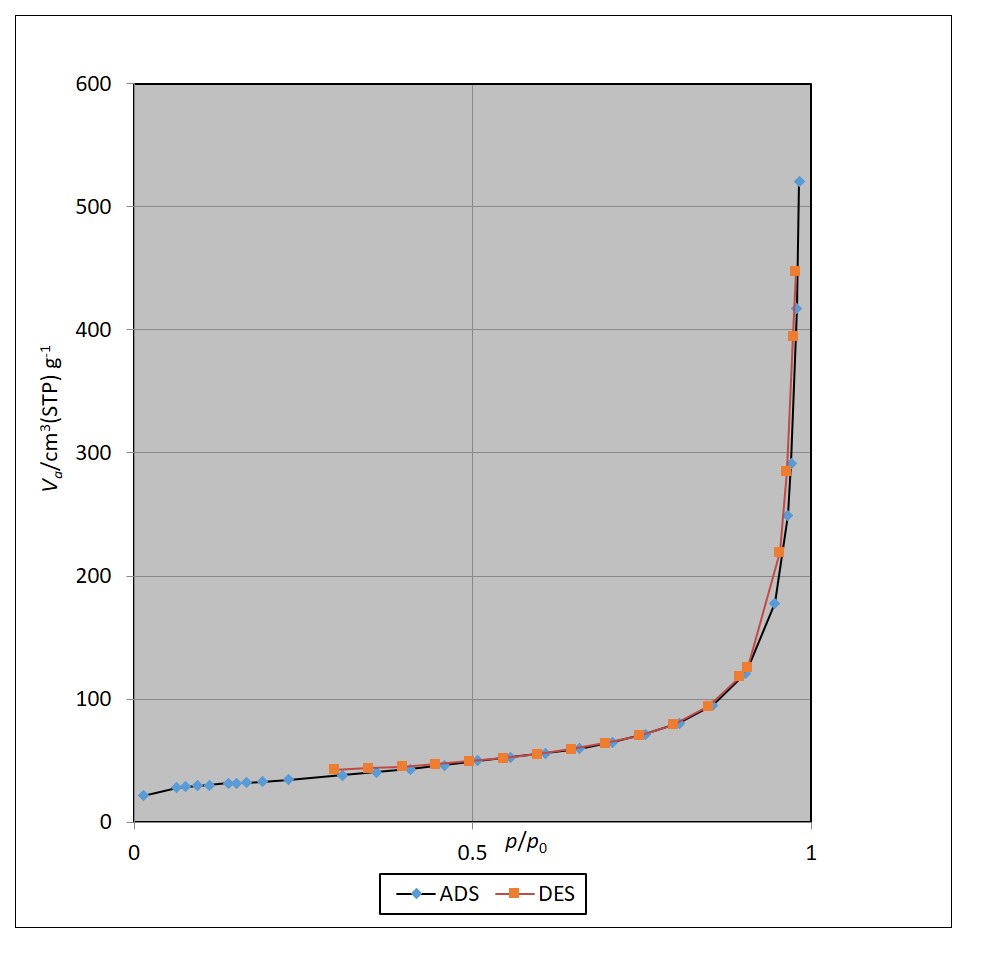


Figure S5. N_2_ Adsorption-desorption isotherms of MIP.


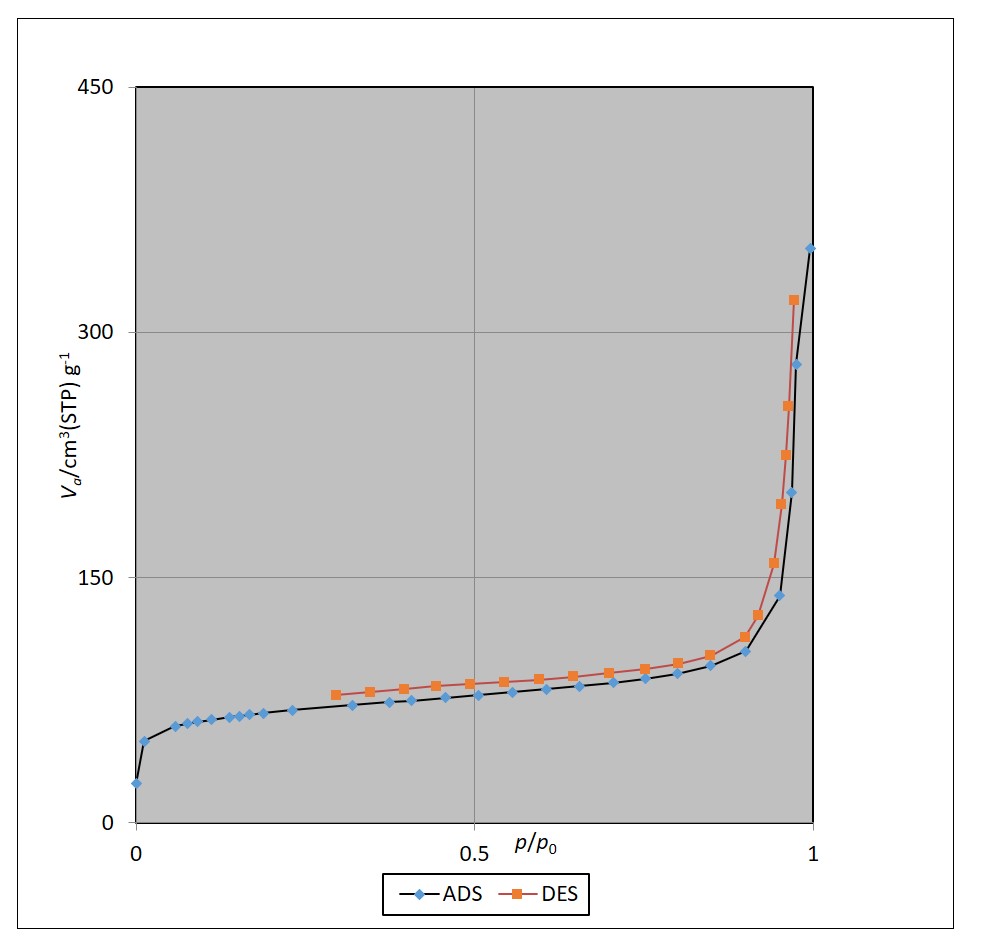


Figure S6. N_2_ Adsorption-desorption isotherms of UiO.


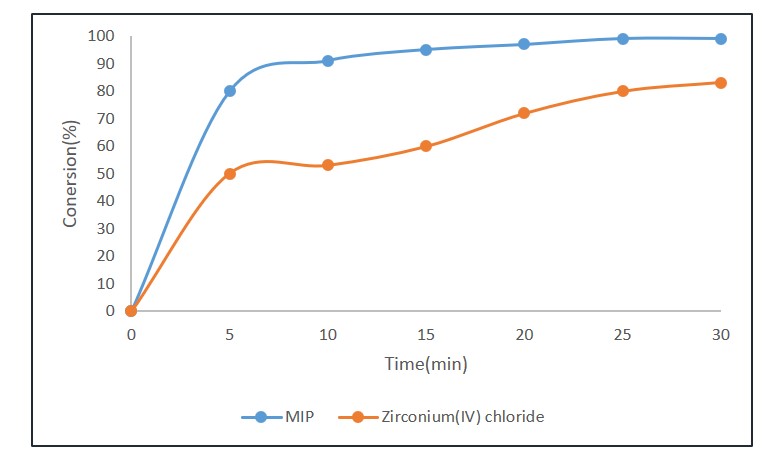


Figure S7. Comparison of zirconium chloride and MIP for ring-opening of styrene oxide with methanol under optimal conditions.

^1^H-NMR (400 MHz, CDCl_3_): δ = 7.29-7.40 (m, 5H), 4.33 (dd, *J* = 8.27, 4 Hz, 1H), 3.60-3.73 (m, 2H), 3.32 (s, 3H), 2.75 (brs, OH, 1H).

^13^C-NMR (100 MHz, CDCl_3_): δ = 137, 127.35, 126.94, 124.93, 84.95, 67.92, 57. [1]


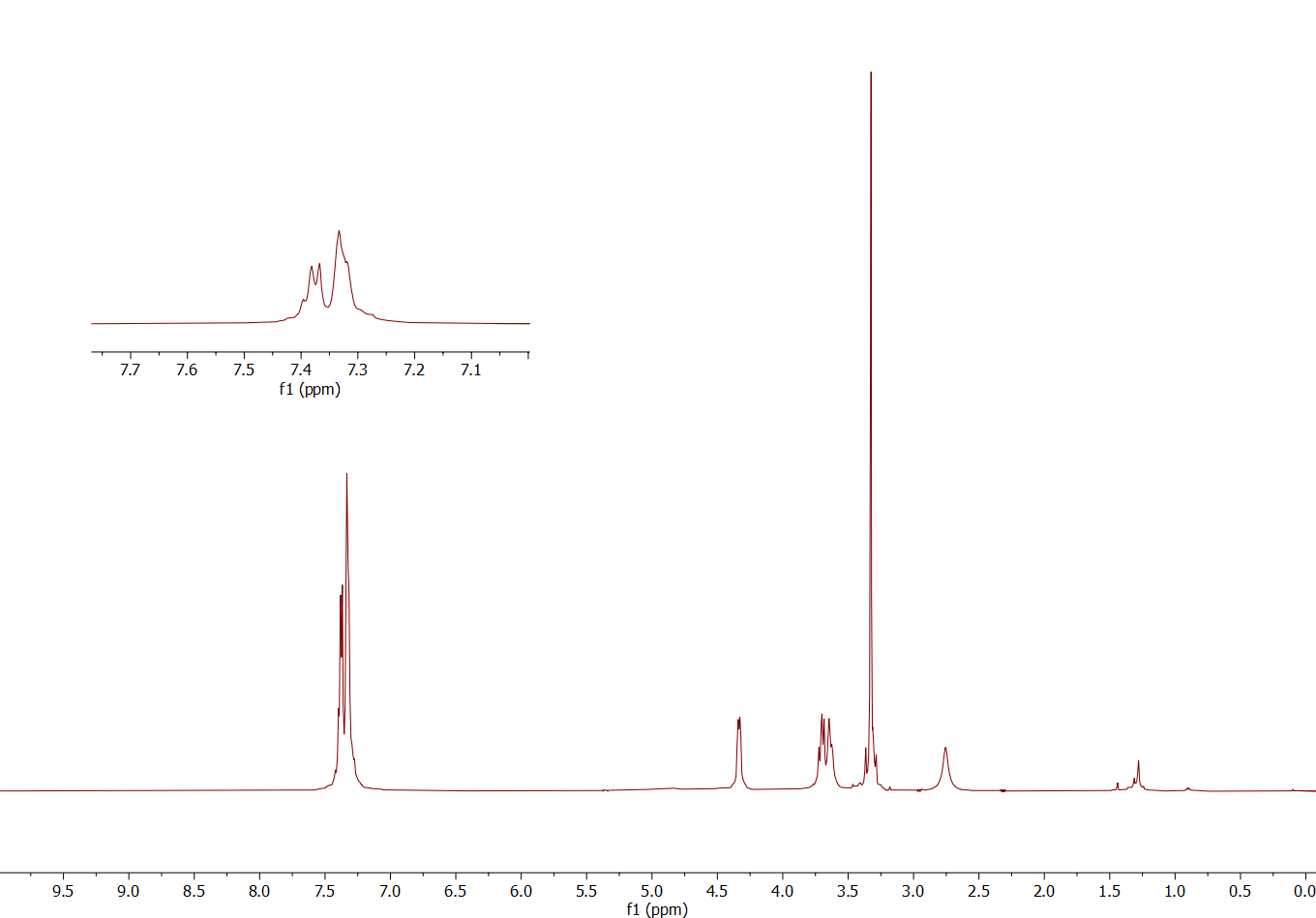


Figure 8S. ^1^H-NMR of 2-methoxy-2-phenylethan-1-ol.


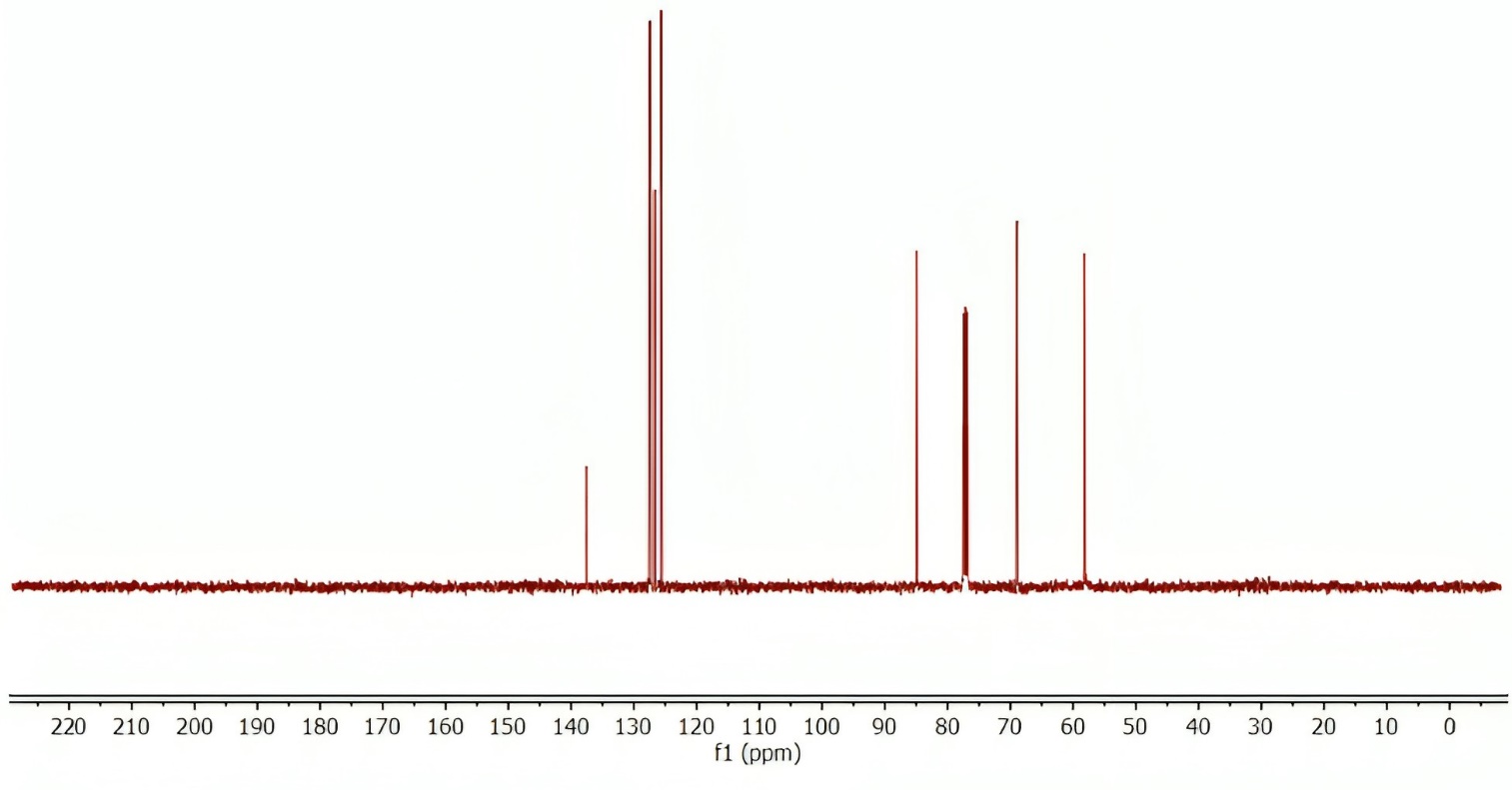


Figure S9. ^13^C-NMR of 2-methoxy-2-phenylethan-1-ol.

^1^H-NMR (400 MHz, CDCl_3_): δ = 7.25-7.42 (m, 5H), 4.34 (dd, *J* = 8.73, 4.44 Hz, 1H), 3.60-3.72 (m, 2H), 3.40-3.55 (m, 2H), 3 (brs, OH, 1H), 1.24 (t, *J* = 7.4 Hz, 3H).

^13^C-NMR (100 MHz, CDCl_3_): δ = 139.07, 129.94, 129.50, 128.12, 84.32, 68.15, 64.60, 15.04. [1]


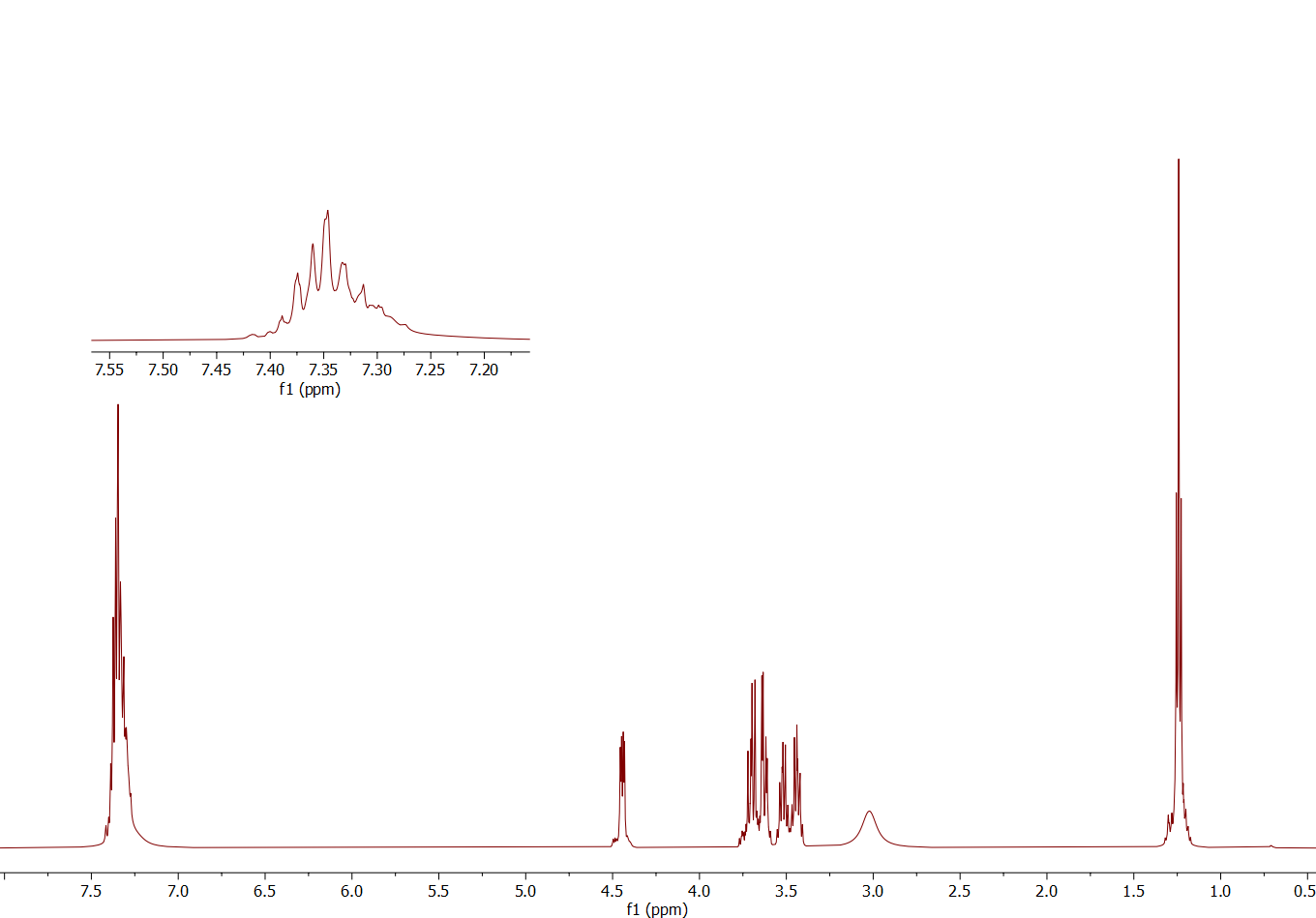


Figure 10S. ^1^H-NMR of 2-ethoxy-2-phenylethan-1-ol.


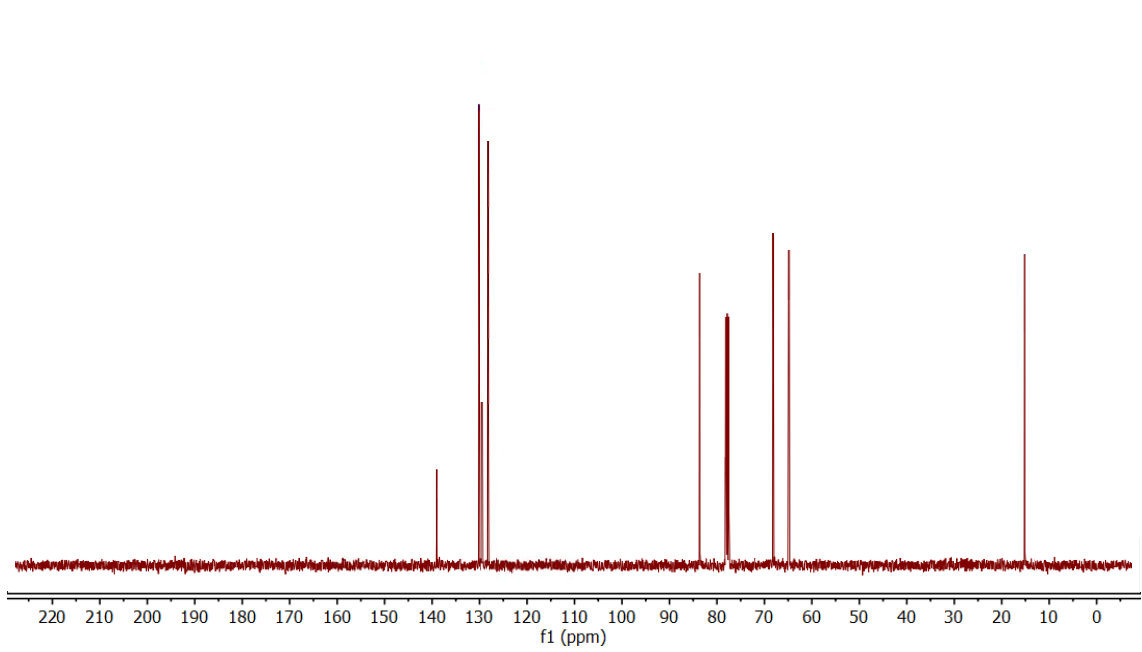


Figure S11. ^13^C-NMR of 2-ethoxy-2-phenylethan-1-ol.

^1^H-NMR (400 MHz, CDCl_3_): δ = 3.92 – 3.74 (m, 1H), 3.5(s, 3H), 3.31 – 3.25 (m, 1H), 1.68 – 1.79 (m, 2H), 1.66 – 1.5 (m, 4H), 1.26 – 1.46 (m, 2H) ppm.

^13^C-NMR (100 MHz, CDCl_3_): δ = 81.1, 67.4, 55.03, 30.04, 25, 23.0, 21.2 ppm.[2]


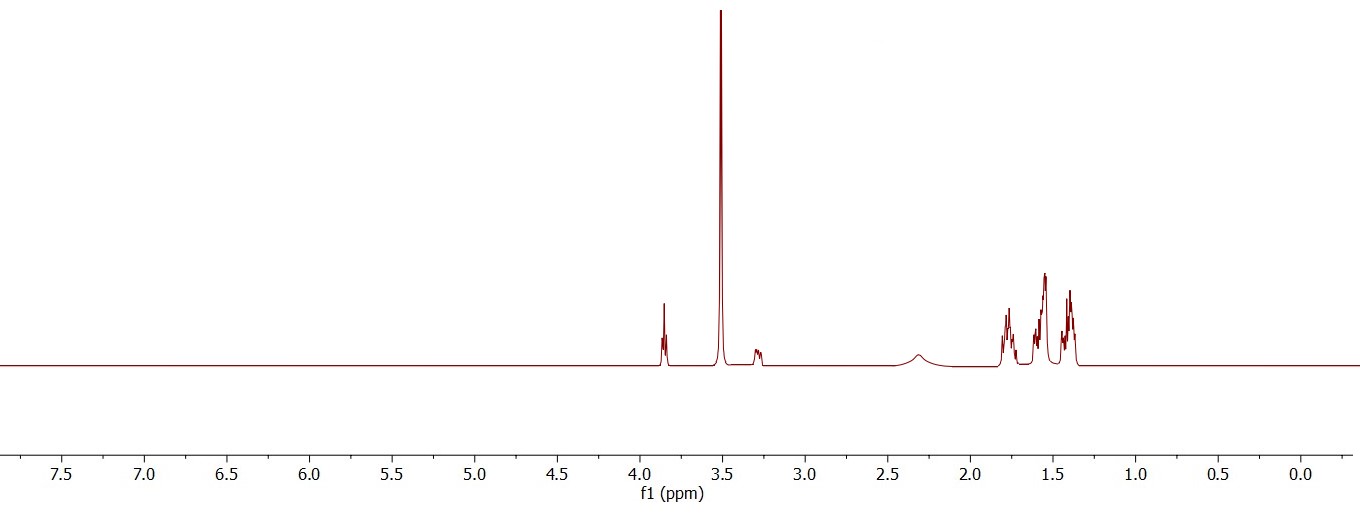


Figure S12. ^1^H-NMR of 2-methoxycyclohexan-1-ol.


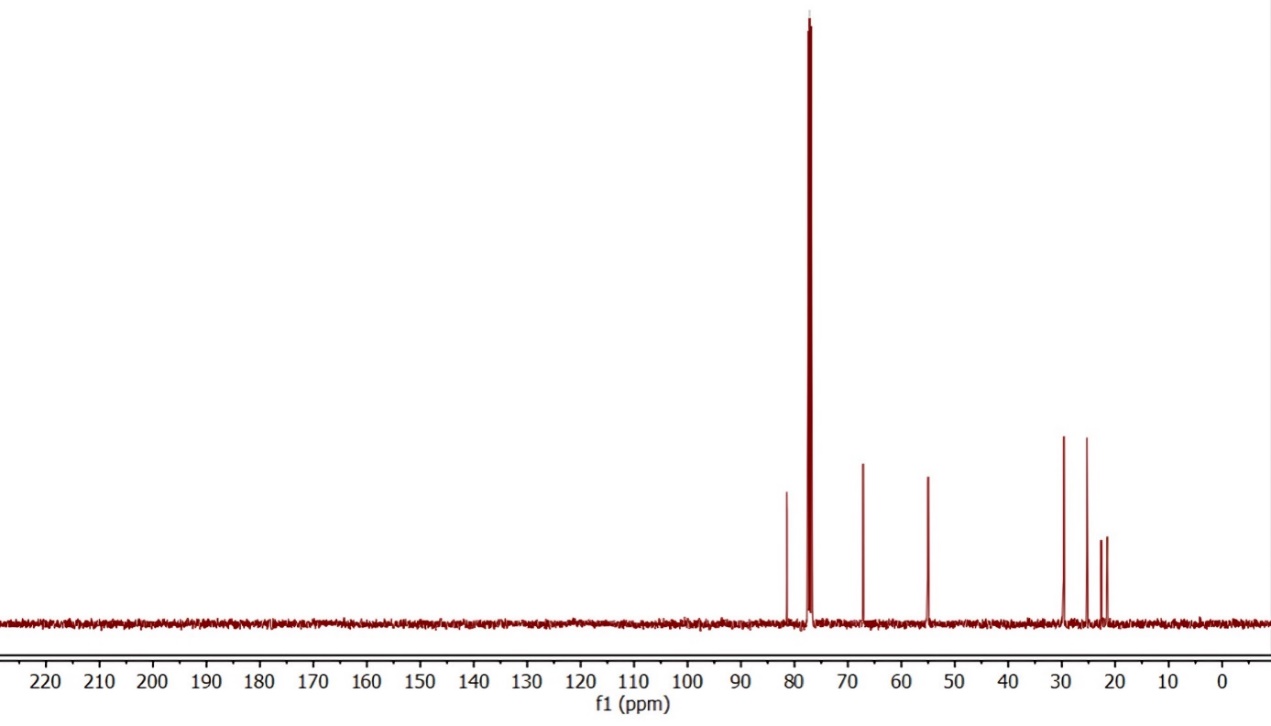


Figure S13. ^13^C-NMR of 2-methoxycyclohexan-1-ol.

^1^H-NMR (400 MHz, CDCl_3_): δ=3.91-3.73 (m, 1H), 3.62-3.33 (m, 3H), 2.75 (bs, OH), 1.88-1.53 (m, 5H), 1.39-1.08 (m, 6H).

^13^C-NMR (100 MHz, CDCl_3_): δ=83.1, 74.4, 61.02,33.4,31.5,24.1,22.2,15.1. [2]


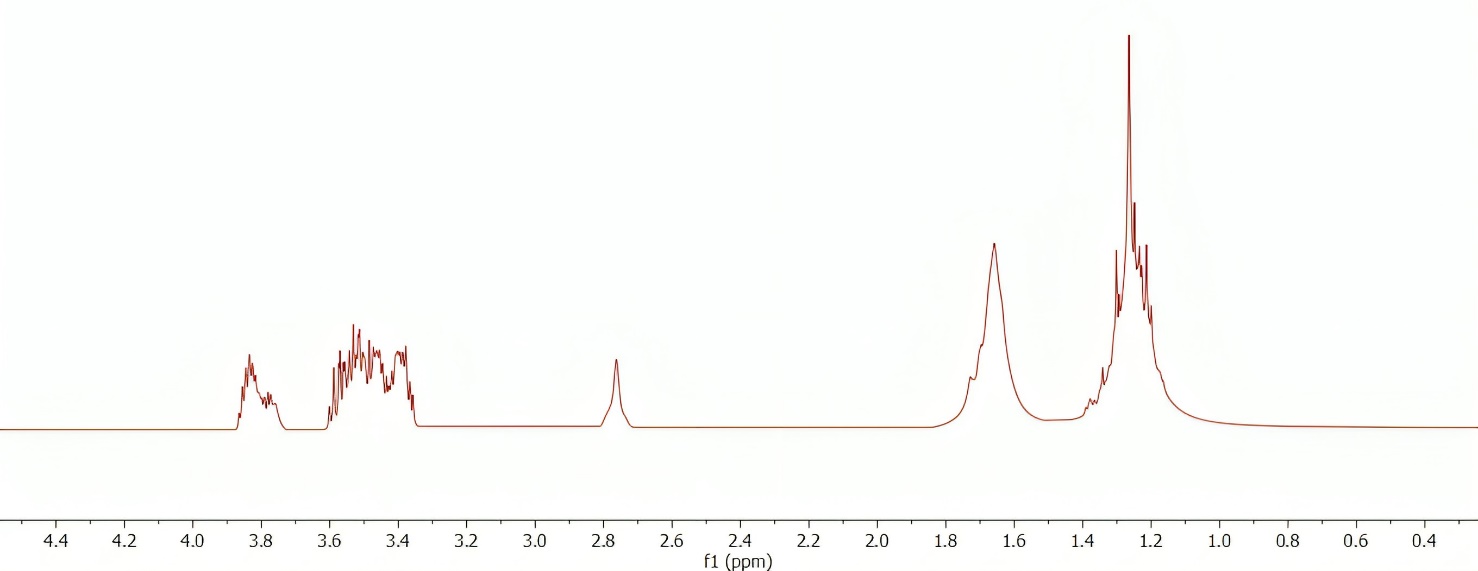


Figure S14. ^1^H-NMR of 2-ethoxycyclohexan-1-ol.


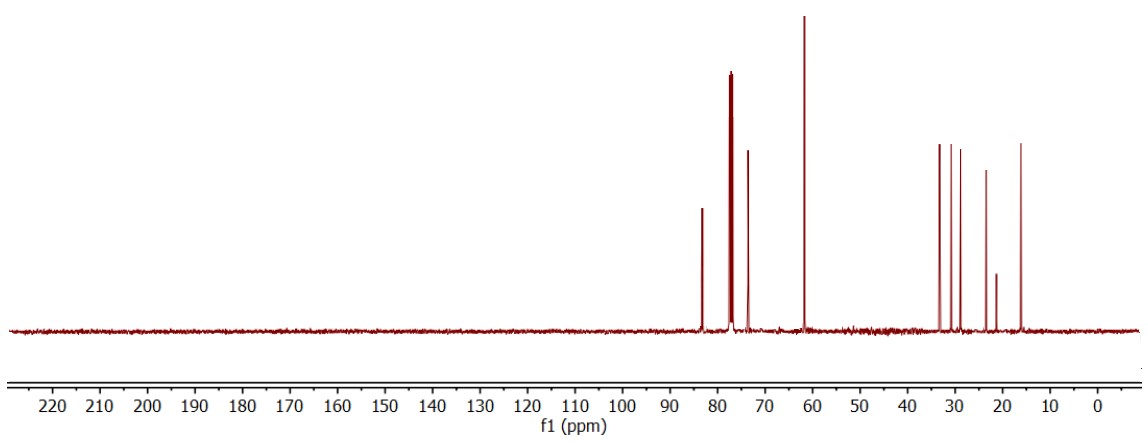


Figure S15. ^13^C-NMR of 2-ethoxycyclohexan-1-ol.

^1^H-NMR (400 MHz, CDCl_3_): δ = 7.46 (m, 5H), 7.22 – 7.27 (m, 2H), 6.84 (t, J = 7.13 Hz, 1H),6.71(d, J= 7.72 Hz, 2H), 4.56 (dd, J =4.06, 1.70 Hz, 1H), 3.94 (dd, J = 11.50, 4.02 Hz, 1H), 3.75 (m, 1H).

^13^C-NMR (100 MHz, CDCl_3_): δ =147.1, 140.1,130.1,129.7,126.2,125.1,119.8,114.7,67.2,57.3. [3]


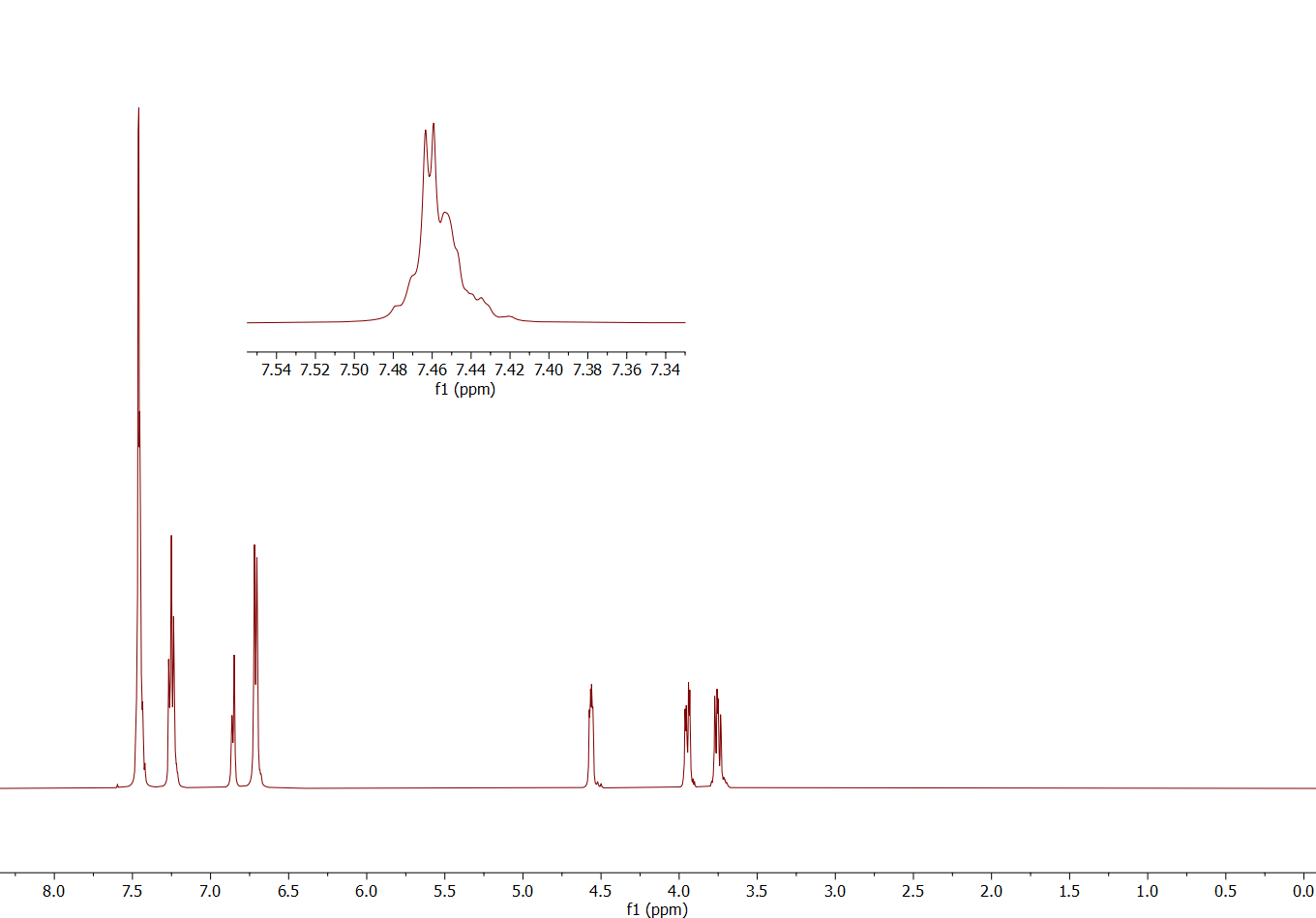


Figure 16 S. ^1^H-NMR of 2-phenyl-2-(phenylamino)ethan-1-ol.


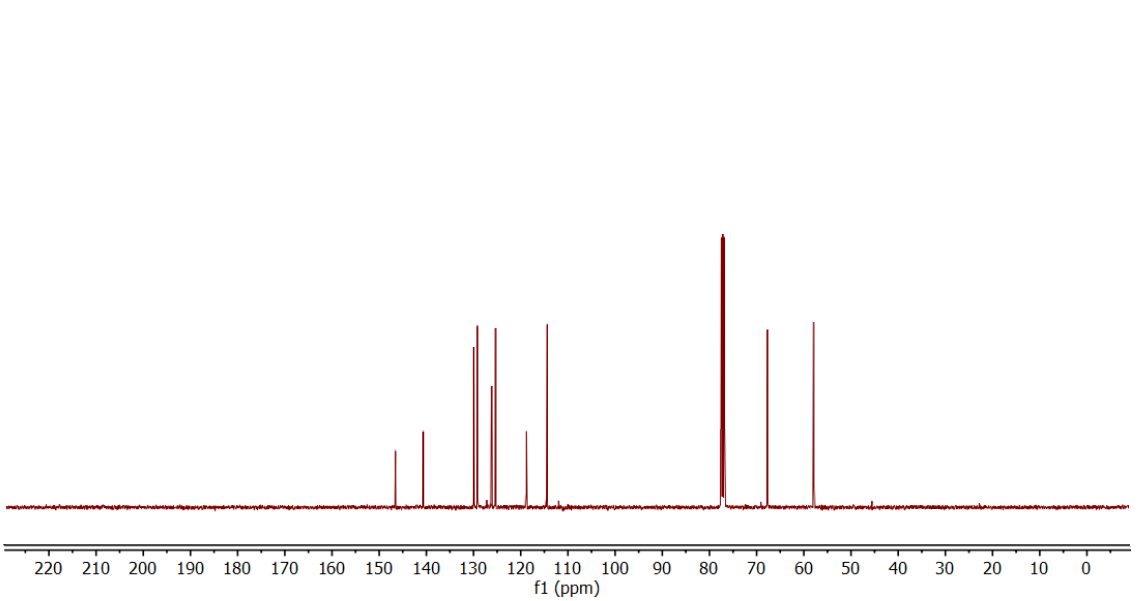


Figure S17. ^13^C-NMR of 2-phenyl-2-(phenylamino)ethan-1-ol.

^1^H-NMR (400 MHz, CDCl_3_): δ =8.75 (d, J = 5 Hz, 2H), 8.15 (d, J = 8.23 Hz, 2H), 7.34 (m, 13H), 6.70(t, J = 8.2 Hz, 4H), 4.74 (dd, J = 10.2, 6.1 Hz, 2H), 4.02 (dd, J = 10.5, 3.9 Hz, 2H), 3.92 (dd, J = 10.9, 6.1 Hz, 2H).

^13^C-NMR (100 MHz, CDCl_3_): δ=146.1,139.8,136.7,130.1,128.2,125.9,125,116.2,114.9,68.1,58.2. [3]


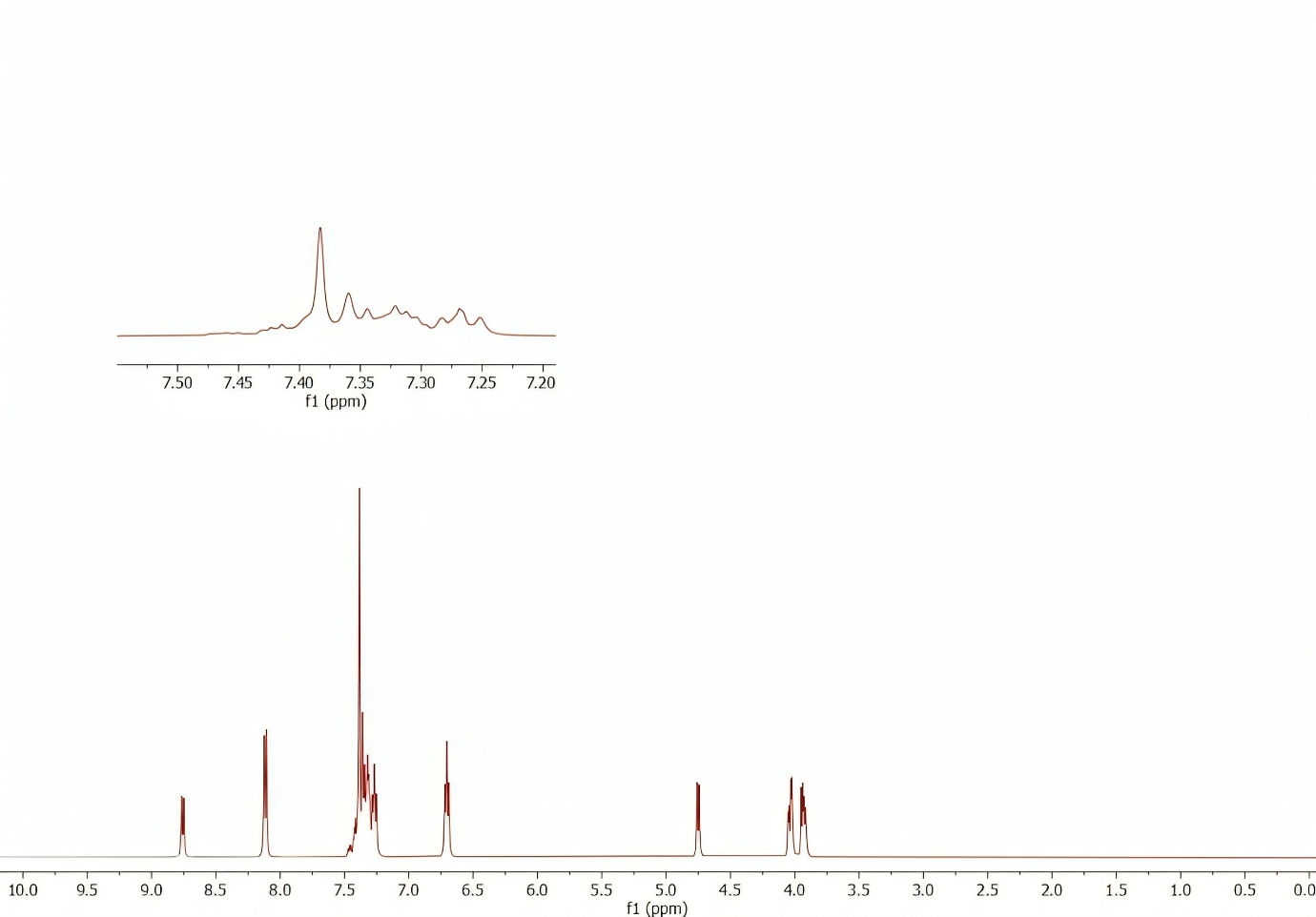


Figure 18 S. ^1^H-NMR of 2-((2-nitrophenyl)amino)-2-phenylethan-1-ol


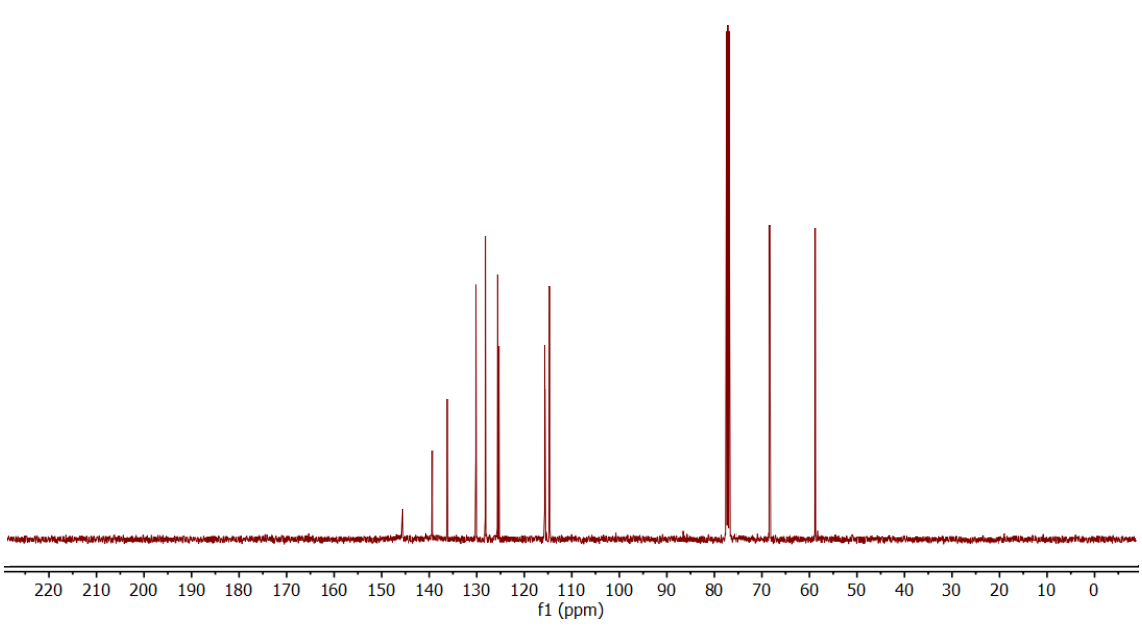


Figure S19. ^13^C-NMR of 2-((2-nitrophenyl)amino)-2-phenylethan-1-ol.

^1^H-NMR (400 MHz, CDCl_3_): δ =8 (d, J = 9.1 Hz, 2H), 7.30 (m, 5H), 6.5 (d, J = 9.3 Hz, 2H), 5.72 (d, J = 4.9 Hz, 1H), 4.59 (d, J =6.43, 1H), 4.03 (dd, J = 11.3, 3.7 Hz, 1H), 3.86 (dd, J = 9.4, 6.4 Hz, 1H), 2.68 (s, 1H).

^13^C-NMR (100 MHz, CDCl_3_):

δ=156.1,155.6,139.6,138.2,131.3,130,129.8,126.3,125.1,124.8,110.1,66.9,58.4

[3]


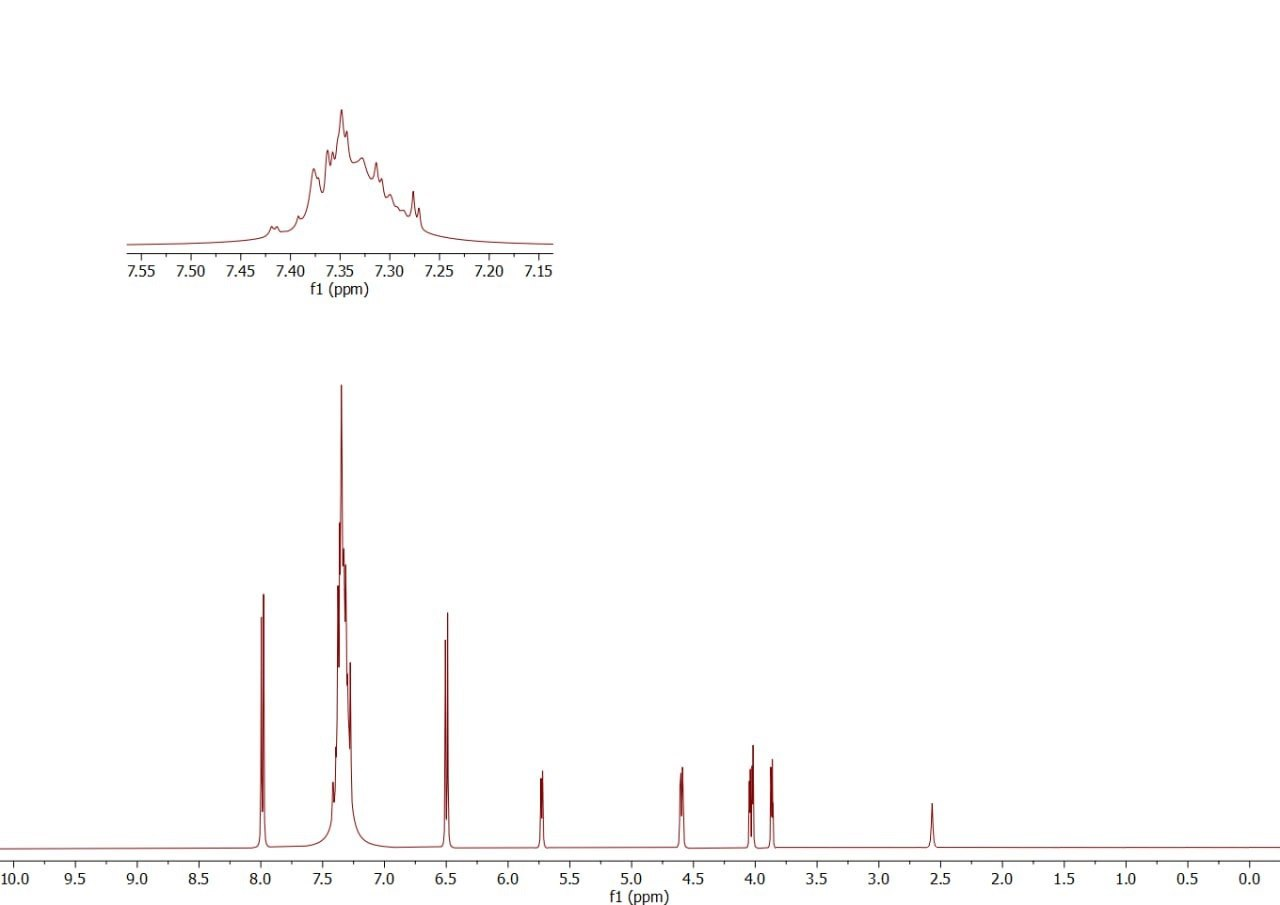


Figure S20. ^1^H-NMR of 2-((4-nitrophenyl)amino)-2-phenylethan-1-ol.


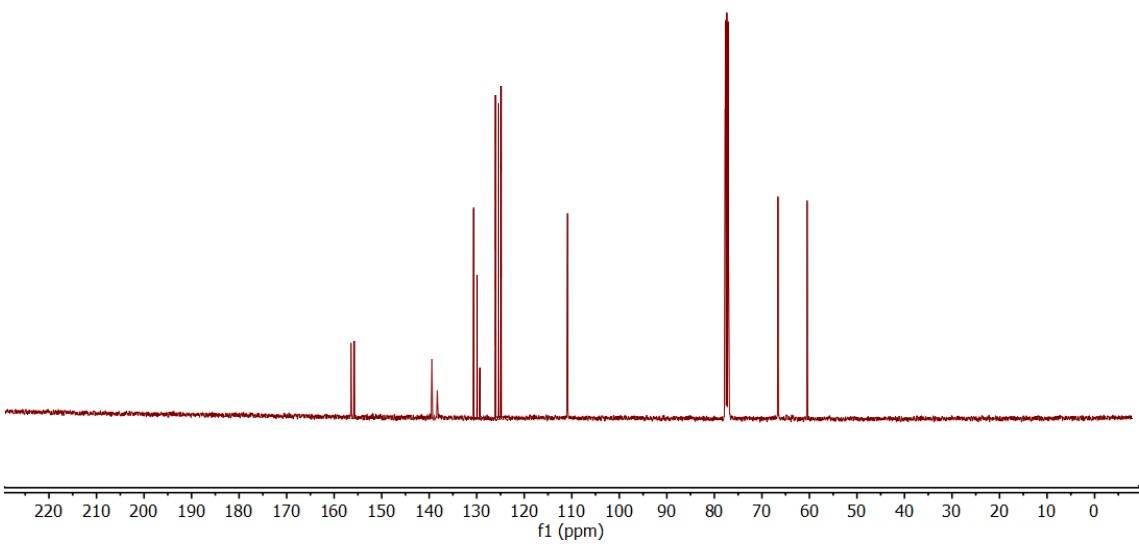


Figure S21. ^13^C-NMR of 2-((4-nitrophenyl)amino)-2-phenylethan-1-ol.

^1^H-NMR (400 MHz, CDCl_3_): δ7.45-7.37(m, 5H),7.045-6.97(m,4H),6.59(d,J=8.47,1H),4.50(ddd, J=6.42,4.22,2.20, 1H),3.92(ddd,J= 6.23 ,4.21,2.02 ,1H),3.76-3.66(m,1H),2.60-2.52(m,2H),1.66-1.56(m,2H),1.46-1.36(m, 2H),1.01(t, J= 7.42 , 3H).

^13^C-NMR(100MHz, CDCl_3_): δ=144.8,143.2,139.8,130.2,129.8,128.6,69.6,63.6,48.3,34.8,33.7,16.4

,12.1


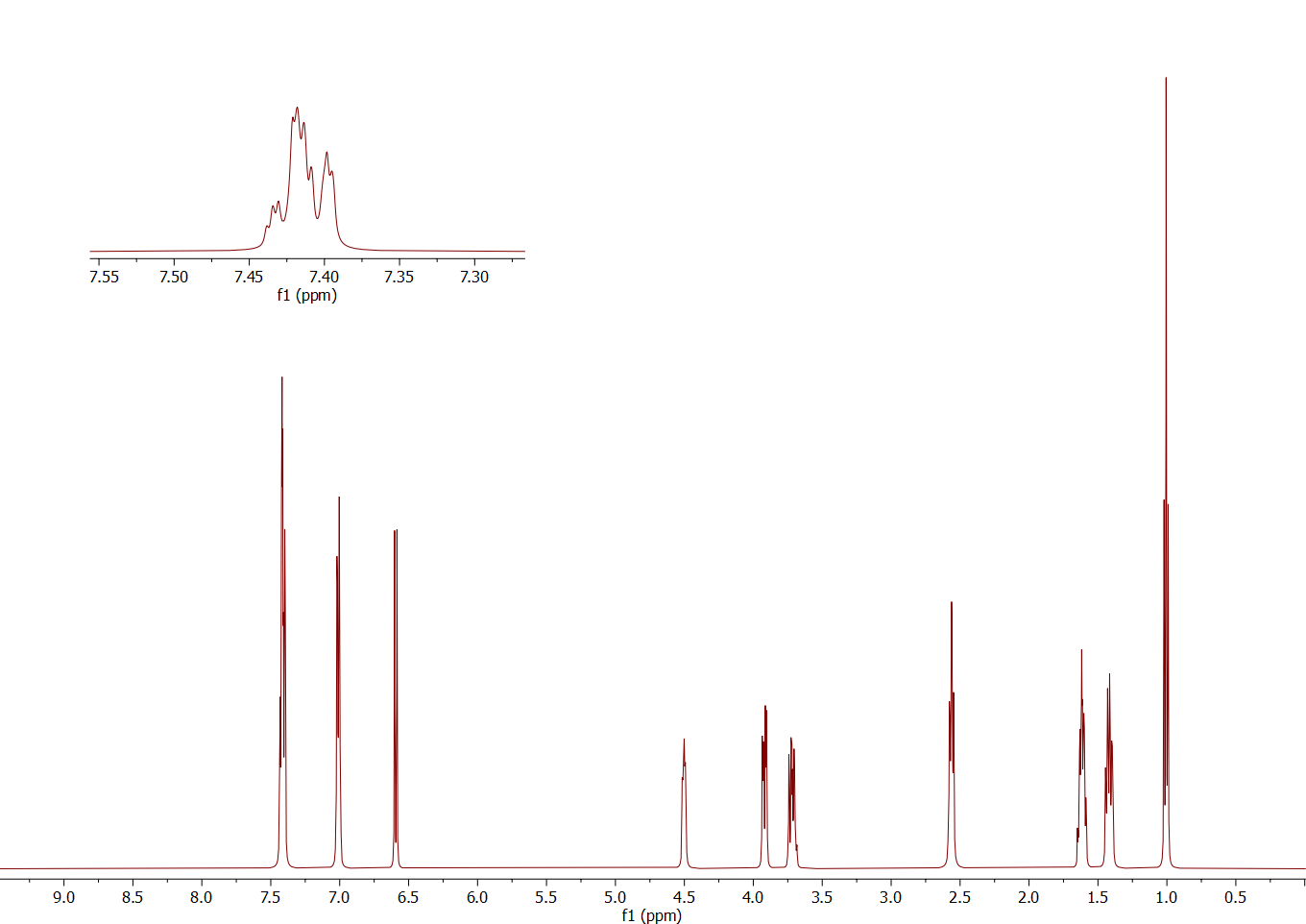


Figure 22 S. ^1^H-NMR of 2-((4-butylphenyl)amino)-2-phenylethan-1-ol.


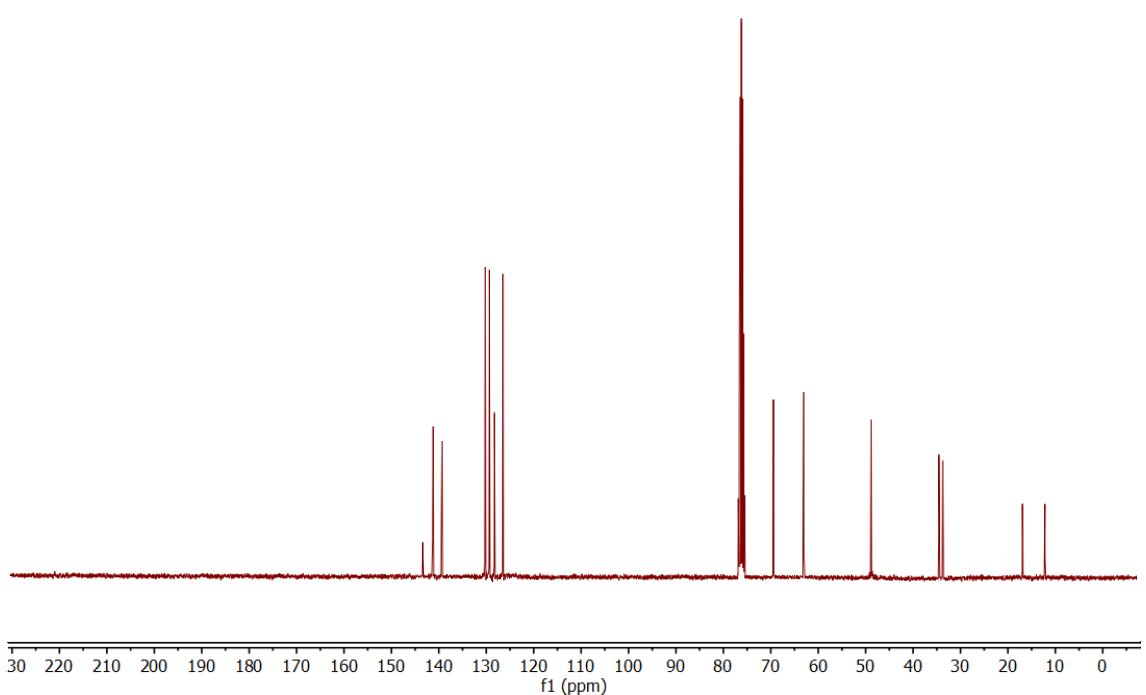


Figure S 23 13C-NMR of 2-((4-butylphenyl)amino)-2-phenylethan-1-ol.

**References**

1. Chaudhary, P. *et al.* Cellulose sulfate: An efficient heterogeneous catalyst for the ring-opening of epoxides with alcohols and anilines. *Synth. Commun.* **51**, 1834–1846 (2021).

2. Talwar, D., Wu, X., Saidi, O., Salguero, N. P. & Xiao, J. Versatile Iridicycle Catalysts for Highly Efficient and Chemoselective Transfer Hydrogenation of Carbonyl Compounds in Water. *Chem. – A Eur. J.* **20**, 12835–12842 (2014).

3. Tyagi, A., Yadav, N., Khan, J., Mondal, S. & Hazra, C. K. Brønsted Acid-Catalysed Epoxide Ring-Opening Using Amine Nucleophiles: A facile access to β-amino alcohols. *Chem. Asian J.* **17**, (2022).
